# Genetic contribution to microglial activation in schizophrenia

**DOI:** 10.1038/s41380-024-02529-1

**Published:** 2024-03-22

**Authors:** Marja Koskuvi, Elina Pörsti, Tristen Hewitt, Noora Räsänen, Ying-Chieh Wu, Kalevi Trontti, Amanda McQuade, Shringaa Kalyanaraman, Ilkka Ojansuu, Olli Vaurio, Tyrone D. Cannon, Jouko Lönnqvist, Sebastian Therman, Jaana Suvisaari, Jaakko Kaprio, Mathew Blurton-Jones, Iiris Hovatta, Markku Lähteenvuo, Taisia Rolova, Šárka Lehtonen, Jari Tiihonen, Jari Koistinaho

**Affiliations:** 1 Neuroscience Center, University of Helsinki, Helsinki, Finland; 2 Department of Physiology and Pharmacology, Karolinska Institutet, Stockholm, Sweden; 3 SleepWell Research Program, Faculty of Medicine, University of Helsinki, Helsinki, Finland; 4 Department of Psychology and Logopedics, University of Helsinki, Helsinki, Finland; 5 Department of Neurobiology & Behavior, UC Irvine, Irvine, CA USA; 6 Sue and Bill Gross Stem Cell Research Center, UC Irvine, Irvine, CA USA; 7 Institute for Memory Impairments and Neurological Disorders, UC Irvine, Irvine, CA USA; 8 Department of Forensic Psychiatry, University of Eastern Finland, Niuvanniemi Hospital, Kuopio, Finland; 9 Department of Psychology and Psychiatry, Yale University, New Haven, CT USA; 10 Mental Health Unit, Department of Public Health Solutions, National Institute for Health and Welfare, Helsinki, Finland; 11 Department of Psychiatry, University of Helsinki, Helsinki, Finland; 12 Institute for Molecular Medicine FIMM, University of Helsinki, Helsinki, Finland; 13 A.I. Virtanen Institute for Molecular Sciences, University of Eastern Finland, Kuopio, Finland; 14 Department of Clinical Neuroscience, Karolinska Institutet, Stockholm, Sweden; 15 Drug Research Program, Division of Pharmacology and Pharmacotherapy, University of Helsinki, Helsinki, Finland

**Keywords:** Neuroscience, Stem cells

## Abstract

Several lines of evidence indicate the involvement of neuroinflammatory processes in the pathophysiology of schizophrenia (SCZ). Microglia are brain resident immune cells responding toward invading pathogens and injury-related products, and additionally, have a critical role in improving neurogenesis and synaptic functions. Aberrant activation of microglia in SCZ is one of the leading hypotheses for disease pathogenesis, but due to the lack of proper human cell models, the role of microglia in SCZ is not well studied. We used monozygotic twins discordant for SCZ and healthy individuals to generate human induced pluripotent stem cell-derived microglia to assess the transcriptional and functional differences in microglia between healthy controls, affected twins and unaffected twins. The microglia from affected twins had increased expression of several common inflammation-related genes compared to healthy individuals. Microglia from affected twins had also reduced response to interleukin 1 beta (IL1β) treatment, but no significant differences in migration or phagocytotic activity. Ingenuity Pathway Analysis (IPA) showed abnormalities related to extracellular matrix signaling. RNA sequencing predicted downregulation of extracellular matrix structure constituent Gene Ontology (GO) terms and hepatic fibrosis pathway activation that were shared by microglia of both affected and unaffected twins, but the upregulation of major histocompatibility complex (MHC) class II receptors was observed only in affected twin microglia. Also, the microglia of affected twins had heterogeneous response to clozapine, minocycline, and sulforaphane treatments. Overall, despite the increased expression of inflammatory genes, we observed no clear functional signs of hyperactivation in microglia from patients with SCZ. We conclude that microglia of the patients with SCZ have gene expression aberrations related to inflammation response and extracellular matrix without contributing to increased microglial activation.

## Introduction

Neuroinflammation is a pathophysiological process involved in various neurological diseases. There is increasing evidence that dysregulation of neuroinflammation and immune response is also associated with neurodevelopmental disorders, including schizophrenia (SCZ) [1, 2]. Microglia are brain resident macrophages indispensable for innate immune system activation and inflammation as a response against invading pathogens or injury-induced products to protect neurons and other glial cells from damage. Although the exact etiology of SCZ remains largely unknown, it is a complex disorder that is thought to develop as the result of the interaction between genetic changes and environmental factors initiated already during embryonal development and continuing through childhood to adolescence and early adulthood [3]. During embryonic development microglia support neurogenesis and modify synaptic network by synaptic pruning in early childhood and adulthood.

Patients with SCZ have upregulation of various markers of neuroinflammation, which has led to the theory of prolonged microglial hyperactivation as a significant contributory factor in SCZ [4]. Postmortem studies have revealed microglial activation in patients with SCZ, detected as increased microglial density based on ionized calcium-binding adapter molecule 1 (IBA1), major histocompatibility complex (MHC) class II, and cluster of differentiation 68 (CD68) immunoreactive cells [5–7]. Consistently, there is evidence for elevated levels of pro-inflammatory cytokines interleukin 6 (IL6), tumor necrosis factor alpha (TNFα), interferon (IFN), and interleukin 1 beta (IL1β) in blood and brain of patients with SCZ [1, 6, 8]. The extended MHC locus has the strongest genetic association with SCZ known so far [9]. The region contains several innate and adaptive immune system related genes, e.g., polymorphic and polygenic antigen-recognition human leukocyte antigen (*HLA*) genes. Due to high MHC locus polymorphism and its involvement in several autoimmune and infectious disorders, it has been hard to locate SCZ-associated genetic variants on this region [10]. Without microglia-specific genetic risk factors, the availability of experimental models to test the impact of SCZ-related microglial function is limited, and the results have been inconclusive.

As the previous studies have suggested hyperactive phenotype in microglia in patients with SCZ, we focused on this study especially on sensitivity to inflammatory stimuli and immune activation. We also studied whether the antipsychotic drug clozapine and two other compounds, minocycline and sulforaphane, have the potential to modify the functions of patient-derived microglia. Clozapine is the most common atypical antipsychotic drug used to treat treatment-resistant SCZ. Minocycline is an antibiotic drug, which has been shown to inhibit microglial activation and synaptic pruning, and thus holds potential for alternative treatment for SCZ [11–14]. Sulforaphane is an antioxidant, which can modulate inflammation-related depression-like behavior in rodents [15, 16]. Clozapine and minocycline have multiple targets, which are not all well-known, while sulforaphane mainly induces NF-E2-related factor-2 (NRF2) activation. Even though it is not completely known how these compounds affect inflammation, previous studies have shown that all three of them can protect cells from LPS-induced immune activation potentially suppressing nuclear factor kappa B (NF-κB) pathway activation [15, 17, 18]. NF-κB is a critical regulator of immune responses and controls the expression of various pro-inflammatory cytokines and acute phase proteins that are increased in the brain in people with SCZ.

We chose to use human induced pluripotent stem cell (hiPSC) derived cell models. We generated hiPSC-derived microglia-like cells (iMGLs) from our well-characterized monozygotic twins discordant for SCZ, i.e., cases with schizophrenia diagnosis and their healthy twins, and unrelated healthy controls, to assess the genetic contribution to differences (1) between affected and unaffected twins and (2) between twins and healthy controls in microglial gene expression and functions. Three of the twin pairs were females and two were males. Similarly, we recruited three female and two male un-related healthy volunteers without history of psychiatric disorders as healthy controls. By using RNA sequencing we show dysregulation of commonly used pan-microglia genes and inflammation-related genes (*AIF1, HLA-DRA*, and *IL1β*) in affected twin microglia. While global gene expression profile was downregulated in both twins compared to healthy individuals, upregulation of 22.6% of the genes was seen only in clinical SCZ cases. Pathways related to extracellular matrix signaling were consistently aberrant in microglia of both affected and unaffected twin. Our test to rescue the expressional changes with applying clozapine, minocycline or sulforaphane gave heterogeneous response in affected twin microglia.

## Materials and methods

### Patient iPSC lines

hiPSCs were generated and characterized in previous study by Tiihonen et al. [19]. Supplementary Tables 1 and 2 summarize the patient cohort and to which experiments they were included. Informed consent was obtained from all participants and all procedures were approved by the Ethics Committee of the Helsinki University Hospital District. Patient iPSCs were cultured in E8 medium (Gibco) on Matrigel (Corning) and splitted 1–2 times a week with 0.5 mM EDTA (Invitrogen) and tested negative for mycoplasma in Neural iPSC (NIPS) unit at the University of Helsinki. hiPSCs from passages 13–25 were used. The used reagents are listed in Supplementary Table 3.

### Microglia differentiation

We used a protocol adapted from McQuade et al. [20] for hiPSC-microglia differentiation. Briefly, hiPSCs were first differentiated to hematopoietic stem cells (HPCs) with StemDiff Hematopoietic kit (StemCell Technologies) according the manufacturer’s instructions. First, hiPSC colonies were de-attached with ReLeSR (StemCell Technologies) and about 20–30 aggregates were seeded per a Matrigel coated 6-well plate in E8 medium with 10 µM ROCK inhibitor (Y-27632, Sigma). On the following day, the StemDiff medium was added onto the wells with well-distributed hiPSC-colonies. The medium was changed every other day 11–13 days until HPCs appeared. Non-adherent HPCs were collected, and 100,000–200,000 cells per well were seeded onto Matrigel-coated well on a 6-well plate in iMGL Base medium consisting of DMEM/F12 (Gibco), 2% ITS-G, 2% B27, 2 mM Glutamax, 1% NEAA, 0.5% N2, 0.5% Penstrep (all from Invitrogen), 5 µg/ml insulin (Sigma) and 400 µM Monothioglycerol (Sigma). In total, 100 ng/ml IL34 (PeproTech), 50 ng/ml TGFβ-1 (PeproTech) and 25 ng/ml M-CSF (Peprotech) were added prior use (= iMGL differentiation medium) and 1 ml of fresh medium was added every other day for 21–26 days. After the first 10–14 days of the differentiation, excess media were collected from the wells leaving only 1 ml on top of the cells. The media were centrifuged 300 × *g* for 4 min to collect the floating microglial precursors that were subsequently replated. To maturate the microglia, the cells were kept for three more days in iMGL maturation medium (iMGL differentiation medium + 100 ng/ml CX3CL1 (PeproTech) and 100 µg/ml CD200 (Biolegend)).

### Drug treatments

The hiPSC-microglia or hiPSC-macrophages were first replated depending on the application and matured for two days. Subsequently, the drugs were added for 24 h in the same medium in the final concentration of 10 µM clozapine (Sigma), 10 µM minocycline (Sigma) or 5 µM D,L-Sulforaphane (Santa Cruz). For the drug studies on LPS-induced inflammation, cells were pre-treated with clozapine, minocycline or sulforaphane for 1 h before adding LPS (Sigma, 100 ng/ml) for 24 h in iMGL maturation medium.

## Results

### Expression of common homeostatic markers of human iMGLs was affected by SCZ

**Fig. 1:**
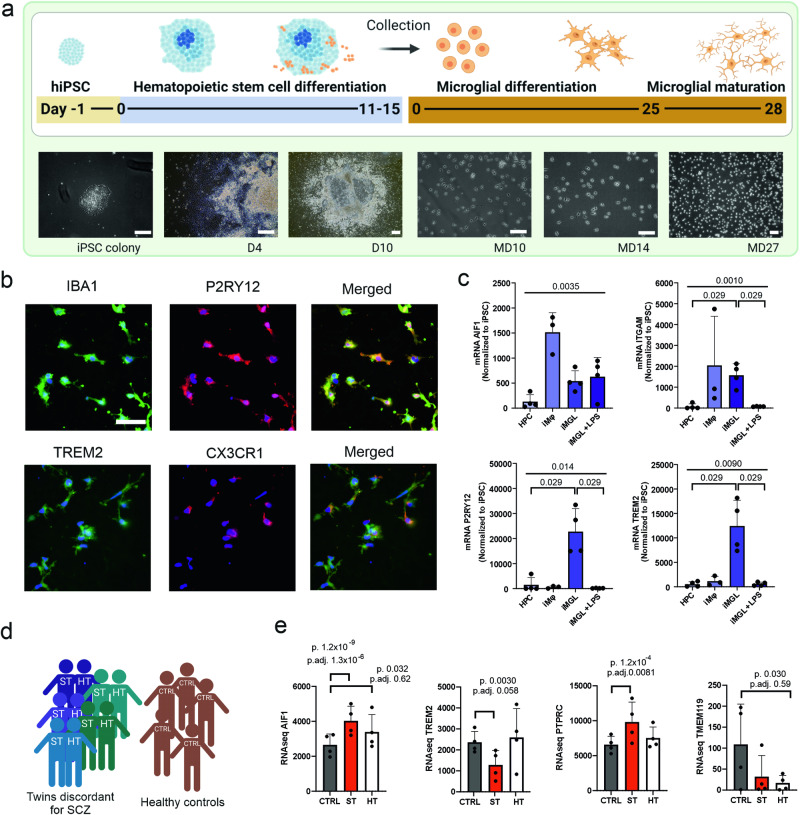
Characterization of human iMGLs derived from monozygotic twins discordant for SCZ. **a** Timeline for iMGL differentiation. Scale bar 50 µm in all. Generated iMGLs expressed (**b**) on protein level pan-macrophage marker IBA1, and microglia-specific markers P2Y12, TREM2 and CX3CR1 (nuclei on blue, scale bar 50 µm), and (**c**) on transcriptomic level *AIF1* (IBA1; *H*(3) = 10.08, *p* = 0.0035), *ITGAM* (CD11b; *H*(3) = 10.93, *p* = 0.001; HPC vs. iMGL *U* = 0, *p* = 0.0286; iMGL vs. iMGL + LPS *U* = 0, *p* = 0.0286), *P2RY12* (*H*(3) = 8.717, *p* = 0.014; HPC vs. iMGL *U* = 0, *p* = 0.0286; iMGL vs. iMGL + LPS *U* = 0, *p* = 0.0286) and *TREM2* (*H*(3) = 9.175, *p* = 0.009; HPC vs. iMGL *U* = 0, *p* = 0.0286; iMGL vs. iMGL + LPS *U* = 0, *p* = 0.0286). Kruskal–Wallis test, Mann–Whitney test, *n* = 3–4 CTRL lines. **d** Illustration of patient cohort created with BioRender. **e** RNA expression of *AIF1*, *TREM2*, *PTPRC* (CD45) and *TMEM119* based on bulk RNA sequencing. CTRL healthy individuals, ST affected twin, HT healthy twin, HPC hematopoietic stem cell, iMφ hiPSC-macrophage, iMGL hiPSC-microglia like cell, iMGL + LPS LPS-treated iMGL.

We used a previously established protocol to generate human iMGLs in 40 days [20] (Fig. 1a). The generated iMGLs had a ramified morphology and expressed pan-macrophage marker IBA1, and microglia-specific markers TREM2, P2RY12 and CX3CR1 (Fig. 1b and Supplementary Fig. 1). iMGLs also upregulated macrophage- and microglia-related genes compared to their intermediate progenitors (hematopoietic progenitor cells, HPCs) and separately generated hiPSC-macrophages (iMφ) (Fig. 1c and Supplementary Fig. 2a). iMGLs responded to 24-h LPS treatment by significant reduction of *ITGAM, TREM2* and *P2RY12* gene expression (*p* = 0.029 in all, Mann–Whitney test). No significant batch differences were discovered (Supplementary Fig. 2b).

We generated iMGLs from a total of five monozygotic twin pairs, where one twin was diagnosed with SCZ (ST) and the other twin was healthy (HT), but had thus a high genetic risk to develop SCZ. Additionally, we generated iMGLs from five non-related healthy individuals (CTRL) (Fig. 1d and Supplementary Table 1). In order to identify disease-related gene expression changes, we performed bulk RNA sequencing for four twin lines and four CTRL lines and compared group expressions to identify differentially expressed genes (DEGs). One of the twin pairs (pair 4) was excluded from RNA sequence due to poor sample quality. The success rate for differentiation was statistically similar for ST iMGL lines (67%, *n* = 18 attempts, *χ*^2^ test), HT iMGL lines (84%, *n* = 19) and CTRL iMGL lines (78%, *n* = 18) (Supplementary Fig. 2d).

We first looked at the expression of common homeostatic microglial markers as their downregulation has been previously implicated in SCZ [21]. None of analyzed markers showed within-pair differences, but the twins differed from CTRLs. *AIF1* (Iba1) was significantly upregulated in both ST and HT iMGLs compared to CTRLs by nominal *p* value (ST *p* = 1.2 × 10^−9^, HT *p* = 0.032) and ST also by Bonferroni corrected *p* value (adj.*p* = 1.3 × 10^−6^) (Fig. 1e). Also *PTPRC* (CD45) was upregulated in ST twins compared to CTRL iMGL (*p* = 0.0012, adj.*p* = 0.0081), while *TREM2* was downregulated in STs (*p* = 0.003) and *TMEM119* downregulated in HTs (*p* = 0.030) compared to CTRL. Other analyzed microglia-specific markers showed no significant group difference, e.g., *P2RY12, CX3CR1, ITGAM*, and *PROS1* (Supplementary Fig. 2c).

### Twins with SCZ had aberrant expression of inflammatory genes and reduced IL6 response to IL1β

**Fig. 2:**
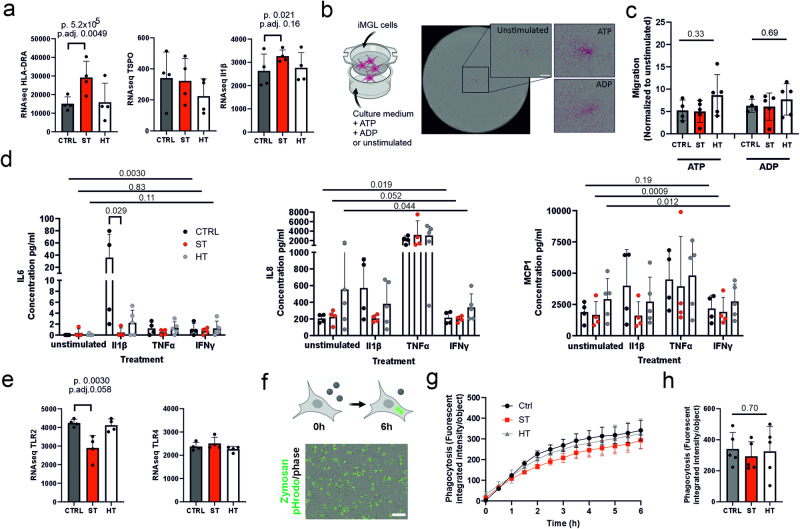
Inflammation, cytokine release and phagocytosis in iMGLs. **a** Gene expression of inflammatory related *HLA-DRA*, *TSPO* and *IL1β* based on RNA sequencing. **b** Representative image after 4-h migration assay. Cells were masked with magenta for analysis. Scale bar 800 µm. **c** The number of migrated cells normalized to unstimulated condition for each group. Performed Kruskal–Wallis test (*H*(2) = 2.371, *p* = 0.3305; *H*(2) = 0.7829, *p* = 0.6927). **d** Release of IL6 (CTRL: *χ*^2^(3) = 10.33, *p* = 0.0030; ST: *χ*^2^(3) = 1.114, *p* = 0.8307; HT: *χ*^2^(3) = 5.864, *p* = 0.1132), IL8 (CTRL: *χ*^2^(3) = 8.400, *p* = 0.0190; ST: *χ*^2^(3) = 7.500, *p* = 0.0517; HT: *χ*^2^(3) = 7.800, *p* = 0.0443) and MCP1 (CTRL: *χ*^2^(3) = 5.100, *p* = 0.1897; ST: *χ*^2^(3) = 11.10, *p* = 0.0009; HT: *χ*^2^(3) = 9.72, *p* = 0.0120) cytokines after 24 h treatment with either 20 ng/ml IL1β, 20 ng/ml TNFα or 20 ng/ml IFNγ. Friedman test and Mann–Whitney test. **e** Gene expression of *TLR2* and *TLR4*. **f** Representative image from phagocytosis after 6 h (Scale bar 100 µm) and (**g**) phagocytosis of pHrodo-zymosan bioparticles. **h** Phagocytosis between the groups after 6 h (*H*(2) = 0.7800, *p* = 0.7027). Kruskal–Wallis test. *n* = 4–5 cell lines.

HLA-DR is one of the commonly used markers for microglial activation in postmortem studies [6, 22, 23]. Expression of *HLA-DRA*, as well as many other HLA class II genes, was upregulated in our ST iMGLs vs. CTRL iMGLs (HLA-DRA *p* = 5.2 × 10^−5^, adj.*p* = 0.0049) (Fig. 2a and Supplementary Fig. 3). The expression of pro-inflammatory cytokine *IL1β* was upregulated in ST vs. CTRL iMGLs (*p* = 0.021). On the other hand, *TSPO*, a controversial marker for inflammation used in PET imaging studies showed no significant alterations [24].

Microglia are extremely motile and survey their environment for injuries and pathogens. In response to ATP and ADP, molecules secreted during neuronal damage, iMGLs showed more than five-fold increased migration (Fig. 2b, c). ST and HT iMGLs responded normally to both ATP and ADP, as the number of migrated iMGL cells did not differ between the groups (Kruskal–Wallis test). Next, we checked whether ST, HT and CTRL iMGLs respond differently to pro-inflammatory cytokines (IL1β, TNFα and IFNγ) measured as induced release of cytokines IL6, interleukin 8 (IL8) and TNFα and chemokine monocyte chemoattractant protein-1 (MCP1) 24 h after exposure. In unstimulated conditions, release of IL6 and TNFα was undetectable. As expected, CTRL iMGLs significantly increased the release of IL6 (*p* = 0.030) and IL8 (*p* = 0.019) after all the cytokine treatments. However, ST iMGLs significantly increased only MCP1 release (*p* = 0.0009, Friedman test). Between ST and CTRL, ST iMGLs had significantly reduced IL6 response to IL1β treatment (*U* = 0, *p* = 0.0286, Mann–Whitney test). These data indicate that ST iMGLs are less responsive to central cell-to-cell pro-inflammatory signals.

Recognition of pathogens is the first step of innate immune activation and thus precedes pro-inflammatory cytokine release. Toll-like receptors (TLRs) detect various viral, bacterial and fungal structures. Interestingly, we observed that *TLR2* expression was significantly downregulated in ST iMGLs compared to CTRL iMGLs (*p* = 0.003), while *TLR4* receptor showed no difference (Fig. 2e). To find out whether the decreased *TLR2* expression of ST iMGLs is reflected as an altered ability to detect pathogens and phagocytose them, we incubated iMGLs with pHrodo-labeled zymosan bioparticles (Fig. 2f and Supplementary Fig. 4), which are recognized through TLR2, TLR6 and non-TLR receptor CLEC7A [25, 26]. A 6-h live imaging ST iMGL showed a slightly reduced phagocytosis of zymosan particles, but the reduction was not statistically significant (*p* = 0.70 Mann–Whitney test) (Fig. 2g, h).

### SCZ iMGLs did not significantly alter neuronal activity in NGN2-neuron co-culture with iMGLs

**Fig. 3:**
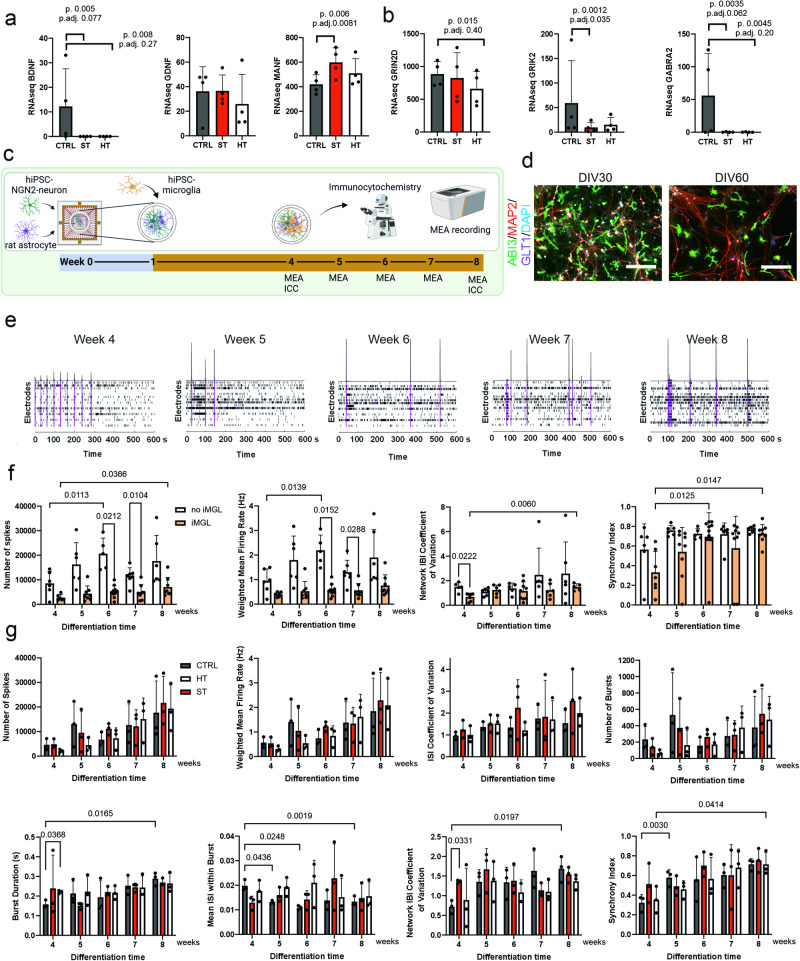
Expression of neurotrophic factors and neural activity in co-culture model. **a** Neurotrophic factor *BDNF*, *GDNF* and *MANF* and (**b**) neurotransmitter receptor *GRIN2D*, *GRIK2* and *GABRA2* gene expression. **c** Timeline for co-culture design and MEA recording. **d** Representative images from CTRL iMGL co-cultures at 30 and 60 days in vitro (DIV) with stainings for NGN2-neurons (MAP2, red), rat astrocytes (GLT1, magenta), iMGLs (ABI3, green), and nuclei (DAPI). **e** Representative raster plots from weekly CTRL iMGL co-culture MEA recordings. Spikes detected on individual electrodes marked with black lines, bursts with blue lines and network bursts with purple boxes. **f** MEA recording results as Number of spikes (95% CI of diff. = −27,769 to −3067, adj.*p* = 0.0212; 95% CI of diff. = −11,671 to −1434, adj.*p* = 0.0104; 95% CI of diff. = −8259 to −331.2, adj.*p* = 0.0366; 95% CI of diff. = −19,980 to −4147, adj.*p* = 0.0113), Weighted mean firing rate (95% CI of diff. = −2.821 to −0.4223, adj.*p* = 0.0152; 95% CI of diff. = −1.418 to −0.07197, adj.*p* = 0.0288; 95% CI of diff. = −2.117 to −0.3807, adj.*p* = 0.0139), Network IBI coefficient of variation (95% CI of diff. = −1.499 to −0.1199, adj.*p* = 0.0222; 95% CI of diff. = −1.181 to −0.4205, adj.*p* = 0.0060), and Synchrony index (95% CI of diff. = −0.6299 to −0.09522, adj.*p* = 0.0125; 95% CI of diff. = −0.6945 to −0.09386, adj.*p* = 0.0147) from co-culture with and without iMGLs. *n* = 5–7 wells for no iMGL and *n* = 6–10 wells from two CTRL iMGL lines. **g** MEA recording results as Number of spikes, Weighted mean firing rate, ISI coefficient of variation, Number of bursts, Burst duration (95% CI of diff. = −0.1197 to −0.007484, adj.*p* = 0.0368; 95% CI of diff. = −0.2053 to −0.05687, adj.*p* = 0.0165), Mean ISI within burst (95% CI of diff. = 0.0004615 to 0.01288, adj.*p* = 0.0436; 95% CI of diff. = 0.002695 to 0.01507, adj.*p* = 0.0248; 95% CI of diff. = 0.005213 to 0.007635, adj.*p* = 0.0019), Network IBI coefficient of variation (95% CI of diff. = −1.574 to −0.3703, adj.*p* = 0.0197; 95% CI of diff. = −1.173 to −0.1141, adj.*p* = 0.0331), and Synchrony index (95% CI of diff. = −0.3168 to −0.1937, adj.*p* = 0.0030; 95% CI of diff. = −0.6867 to −0.03380, adj.*p* = 0.0414) from ST, HT and CTRL iMGL co-cultures. *n* = 3 lines per group. Dunnett’s (timepoints compared to week 4 timepoint) or Tukey’s (comparison between with/without iMGL groups) multiple comparisons tests were used for significance. CTRL healthy individuals, ST affected twin, HT healthy twin.

In addition to neuroinflammation regulation, promotion of neurogenesis and synaptogenesis by neurotrophic factor secretion is another important function of microglia [27]. Especially brain-derived neurotrophic factor (BDNF) released by microglia enhances learning-related synapse formation [28]. BNDF is also one of the most considered biomarkers for SCZ, which has been found to be reduced in plasma samples in SCZ [29]. Interestingly, *BDNF* was significantly less expressed in both ST and HT iMGLs compared to CTRL iMGLs (*p* = 0.005 and *p* = 0.008, respectively) (Fig. 3a). iMGLs expressed also glial cell line-derived neurotrophic factor (*GDNF*), which has been reported to show de novo expression in microglia/macrophages upon neuroinflammation or injury [30], but its expression was similar in all three groups (Fig. 3a). Another neurotrophic factor that showed altered expression in ST iMGLs was mesencephalic astrocyte-derived neurotrophic factor (*MANF*), which in turn was upregulated in ST iMGLs vs. CTRL (*p* = 0.006, adj.*p* = 0.008). MANF has been reported to induce anti-inflammatory microglia polarization after endoplasmic reticulum-associated stress [31]. Microglia express different neurotransmitter receptors for communicating with neurons and glia [32–34]. We found that especially N-methyl-D-aspartate (NMDA)-receptor gene *GRIN2D* was highly expressed in iMGLs and showed downregulation in HT vs. CTRL (*p* = 0.015) (Fig. 3b).

Other neurotransmitter genes, such as kainate receptor *GRIK2* and GABA-receptor *GABRA2* were expressed only in low level, but were significantly downregulated in ST and HT compared to CTRL iMGLs (ST *GRIK2* *p* = 0.0012; ST *GABRA2* *p* = 0.0035, and HT *GABRA2* *p* = 0.0045). Even though no microglial complement pathway genes (C1Q and C3) were expressed differently in ST or HT iMGLs (Supplementary Fig. 5), the reduction in neurotrophic factors and neurotransmitter expression suggests reduced potential for synaptic remodeling in microglia from patients with SCZ.

In order to investigate if the reduction in neurotrophic factor expressions in iMGLs from patients with SCZ leads to altered neuronal activity, we designed a tri-culture system with doxycycline-inducible Neurogenin 2 (NGN2)-expressing hiPSC-cortical neurons, rat astrocytes and iMGLs (Fig. 3c). Astrocyte, neuron, and microglial survival were verified by stainings with cell-type specific markers in 30 and 60 days old co-cultures (Fig. 3d and Supplementary Fig. 6). To follow functional maturation of the neurons in co-cultures, we used multi-electrode array (MEA) to detect neural activity and synchronicity. We were able to reliably detect network-level activity after 4 weeks in co-culture and follow it until 8 weeks (Fig. 3e and Supplementary Fig. 7). First, we tested how adding iMGLs changes neural firing by comparing co-cultures without iMGLs with two CTRL line iMGL co-cultures. Including iMGLs into co-culture system significantly reduced neural activity at 6- and 7-week time points (reduced number of spikes *p* = 0.021 and *p* = 0.01, lower weighted mean firing rate *p* = 0.015 and *p* = 0.029, Tukey’s multiple comparisons test) and the activity significantly increased by time in co-cultures without microglia (number of spikes 4 vs. 6 weeks *p* = 0.011, and mean firing rate 4 vs. 6 *p* = 0.01; Dunnett’s multiple comparisons test; Fig. 3f). On the other hand, Network IBI (inter-network burst interval) coefficient of variation for network burst rhythmicity and Synchrony index for spiking synchronicity were lower in iMGL containing co-cultures than without iMGLs (Network IBI coefficient of variation 4 weeks *p* = 0.022) and these measures increased over time until no differences were detectable between the co-cultures at later time points (Network IBI coefficient of variation 4 vs. 8 weeks *p* = 0.006, Synchrony index 4 vs. 6 weeks *p* = 0.013, 4 vs. 8 weeks *p* = 0.015). Additional data from burst and network burst metrics are collected in Supplementary Fig. 8. Thus, NGN2 neurons were firing less with iMGL co-cultures and it took longer for them to establish mature network-level activity.

Next, we compared the co-cultures with three female CTRL, ST or HT iMGL lines (Fig. 3g and Supplementary Fig. 9). Overall, there were no differences in neural spiking activity (number of spikes, weighted mean firing rate and ISI (inter-spike interval) coefficient of variation) between neurons co-cultured with CTRL, HT, and ST-derived iMGLs. The number of bursts was not altered either, but the burst duration was significantly increased in HT vs. CTRL iMGL co-cultures at 4-week time point (*p* = 0.037), and between 4 and 8 weeks in CTRL iMGLs (*p* = 0.017), but not in ST or HT iMGL co-cultures. Mean ISI within bursts significantly decreased over time in CTRL iMGLs indicating increased spiking within bursts (in CTRLs 4 vs. 5 weeks *p* = 0.047, 4 vs. 6 weeks *p* = 0.025, 4 vs. 8 weeks *p* = 0.002). The only significant difference between ST iMGL co-cultures compared with CTRL or HT co-cultures was an increase between ST and CTRL iMGLs in Network IBI coefficient of variation at the 4-week time point (*p* = 0.033). This indicates greater irregularity in neuronal network bursting in ST iMGL containing cultures. No significant changes were detected in ST iMGL co-culture neural network activity compared to the 4-week time point, but bursting in CTRL and HT iMGL co-cultures slightly improved over time. ST iMGL cultures gave more mature firing earlier than CTRL and HT co-cultures, but we did not see changes in ST iMGLs over time. However, before 4 weeks of age only a small number of electrodes per well showed activity, hence it was not reliable to use earlier time point. We conclude that even though the electrophysiological properties of NGN neurons were slightly differentially affected by ST, HT and control iMGLs, the iMGL co-culture system did not produce clear evidence for the impact of ST iMGLs on neuronal activity.

### Global downregulation of genes was associated with increased risk to develop SCZ and upregulation of MHC region to clinical manifestation of SCZ

**Fig. 4:**
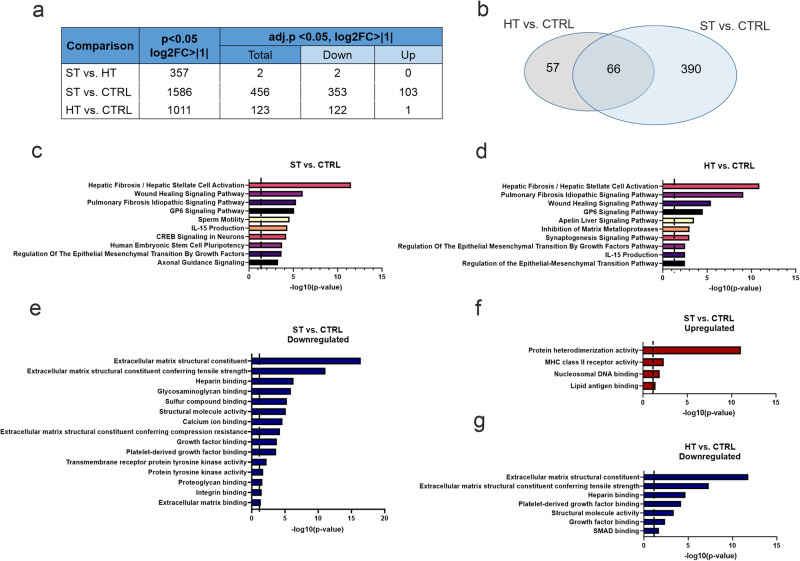
iMGL expression profile and pathway analysis. **a** Overview of differentially expressed genes (DEGs) in different comparisons. **b** Venn diagram of shared DEGs between HT vs. CTRL and ST vs. CTRL. Ingenuity Pathway Analysis (IPA) canonical pathways (**c**) from ST vs. CTRL and (**d**) HT vs. CTRL comparisons. Cutt-offs: Fold change > abs 2, adjusted *p* < 0.05. **e** Downregulated and (**f**) upregulated Gene Ontology (GO) Molecular function terms from ST vs. CTRL comparison and (**g**) downregulated terms from HT vs. CTRL comparison. CTRL healthy individuals, ST affected twin, HT healthy twin. RNAseq *n* = 4 lines in each group.

We further examined comprehensively the DEGs between the study groups (Fig. 4a). While the ST twins differed significantly from the HT twins only by 2 DEGs (adj.*p* < 0.05, abs log2FC > 1), we found more expression changes related to clinical illness (ST vs. CTRL, 456 DEGs) than increased risk to develop SCZ (HT vs. CTRL, 123 DEGs) (Supplementary Data file 1–3). Most of the SCZ-related gene expression changes were reductions, as 77.4% of the DEGs in ST vs. CTRL and 99.2% in HT vs. CTRL were downregulated. These comparisons overlapped by 66 DEGs, which is 53.7% of altered expression changes in the HT vs. CTRL comparison and 14.5% in the ST vs. CTRL comparison (Fig. 4b).

Next, we used Ingenuity Pathway Analysis (IPA) to identify canonical pathways related to DEGs in ST or HT vs. CTRL comparisons in iMGLs (adj.*p* < 0.05 and abs log2FC > 1 threshold for DEGs). Both twin comparisons to CTRL group shared the first four pathways, which together with Wound Healing Signaling Pathway and Pulmonary Fibrosis Idiopathic Signaling Pathway contained Hepatic Fibrosis/Hepatic Stellate Cell Activation (−log10(*p* value) = 11.5 in ST vs. CTRL and 10.9 in HT vs. CTRL) and GP6 Signaling Pathway (−log10(*p* value) = 5.1 in ST vs. CTRL and 4.5 in HT vs. CTRL). These pathways include genes encoding extracellular matrix molecules and have been identified in previous studies with the same cohort to be involved in hiPSC-neuron and hiPSC-astrocyte dysregulation in ST [35, 36] (Fig. 4c, d and Supplementary Data files 4 and 5).

In order to study down- and upregulated DEGs separately, we used additionally Gene Ontology (GO) term analysis (Supplementary Data file 6–8). Downregulated GO Molecular function terms were similar between ST vs. CTRL and HT vs. CTRL (Fig. 4e, g). The most significant functions were Extracellular matrix structural constituent (−log10(*p* value) = 16.4 in ST vs. CTRL and 11.8 in HT vs. CTRL), Extracellular matrix structural constituent conferring tensile strength(−log10(*p* value) = 11.1 in ST vs. CTRL and 7.3 in HT vs. CTRL), and Heparin binding (−log10(*p* value) = 6.3 in ST vs. CTRL and 4.7 in HT vs. CTRL) terms. These pathways shared several collagen genes.

In turn, the upregulated GO terms in STs compared to CTRLs were related to Protein heterodimerization (−log10(*p* value) = 11.0) and MHC class II receptor activities (−log10(*p* value) = 2.3) (Fig. 4f). The first functional term contains several histone modification genes of which Clustered Histone genes *H3C12, H2BC17, H2AC13, H3C10*, and *H3C11* (5/18 DEGs) belong to a small cluster of histone genes in chromosome 6p22.1, which is in close proximity of MHC region (Supplementary Data file 7) [37]. Also nine out of 18 DEGs belonged to a large histone cluster in 6p22.2. MHC class II receptor activity term consists of HLA-genes (*HLA-DQA2* and *HLA-DQB2* genes in Supplementary Fig. 3). Thus, clinical manifestation of SCZ is associated with activation of chromosome 6 MHC region in iMGLs.

### Heterogeneous response to different drug treatments in twins with SCZ

**Fig. 5:**
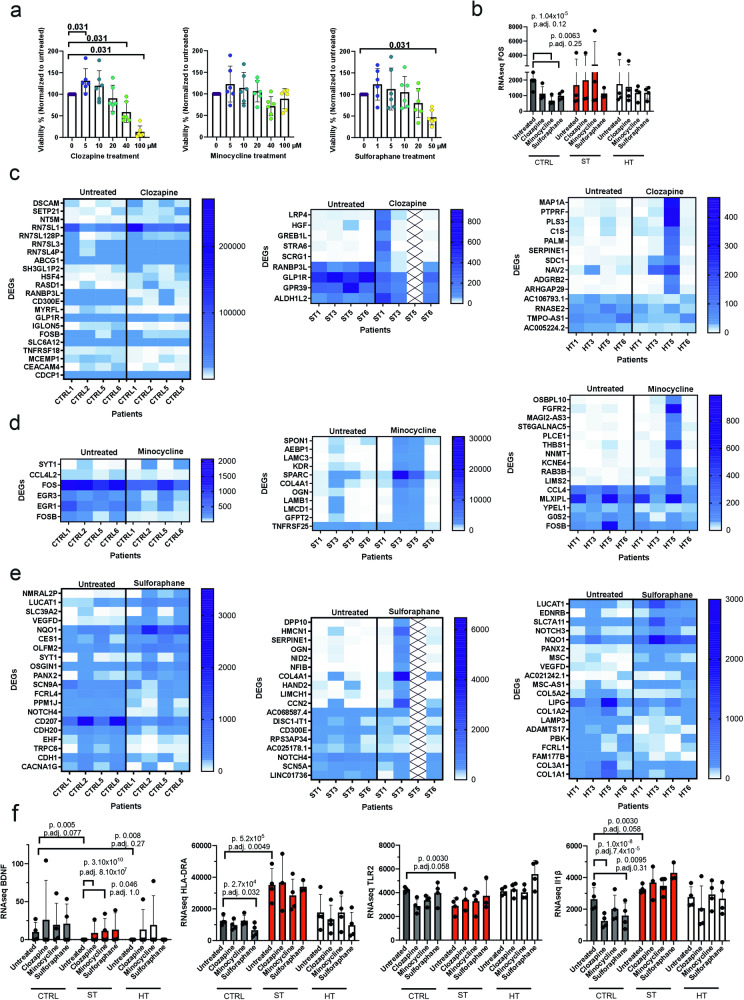
iMGL responses to different drug treatments. **a** Viability of iMGLs after 24-h treatment with clozapine (*W* = −21.00, *p* = 0.0313 all), minocycline and sulforaphane (*W* = −21.00, *p* = 0.0313). Tested with four independent experiments with two control, two HT and two ST lines (*n* = 6 lines) (Wilcoxon test). **b** NFκB pathway downstream gene *FOS*. Heatmaps of CTRL, ST and HT line gene expression changes after (**c**) 10 µM clozapine (**d**) 10 µM minocycline and (**e**) 5 µM sulforaphane treatments. No data from ST5 patient after clozapine and sulforaphane. **f** Gene expression differences after drug treatments in *BDNF, HLA-DRA, TLR2* and *IL1β*. RNAseq *n* = 3–4 lines in each group. CTRL healthy individuals, ST affected twin, HT healthy twin.

As STs showed specific dysregulations to several SCZ-associated genes, we tested if these transcriptomic changes can be rescued either with clozapine, an efficient atypical antipsychotic drug, minocycline, a tetracycline antibiotic proposed also as treatment of negative symptoms in SCZ due to its anti-inflammatory properties, and sulforaphane, an organosulfur isothiocyanate antioxidant with histone deacetylase inhibitory properties proposed for treatment of cognitive functions for patients with SCZ. We selected the drug concentration range from previously used cell culture studies with primary murine microglia or BV2 cell line microglia [12, 17, 38], and tested the suitable concentration by viability assay. Clozapine and sulforaphane had dose-dependent decreased effect on cell viability after 24 h, while minocycline showed no clear toxicity up to 100 µM concentration (Fig. 5a).

We tested the drug effect on phagocytosis, as minocycline has been previously shown to reduce phagocytosis of synaptic material dose-dependently in monocyte-derived microglia [12]. Phagocytosis was significantly increased after 24-h treatment with 10 µM sulforaphane, but no significant changes were discovered after clozapine or minocycline treatment (Supplementary Fig. 10a, c). We repeated the experiment with iMφs, but again only sulforaphane significantly increased phagocytosis of zymosan, and the highest used concentration of clozapine and sulforaphane were toxic and caused significant reduction in phagocytosis (Supplementary Fig. 10b, d). As we could not detect any response in phagocytosis of zymosan after minocycline treatment, this drug might inhibit only complement system mediated synapse phagocytosis as previously reported [12].

We continued using drug concentrations which did not affect viability (clozapine 10 µM, minocycline 10 µM and sulforaphane 5 µM), and performed RNA sequencing for drug treatments as well. All of the three drugs have been suggested to suppress NF-κB pro-inflammatory pathway activation as one beneficial mechanism. NF-κB downstream transcription factor *FOS* was significantly downregulated in CTRL iMGLs after 24-h minocycline and sulforaphane treatments, but not after clozapine treatment (Fig. 5b). However, the same downregulation was not seen in ST or HT twins untreated vs. treated. None of the drugs had any significant effect on IL8 and MCP-1 release (Supplementary Fig. 10e–g).

As clozapine inhibits NF-κB activation by inhibiting Akt pathway and sulforaphane promotes anti-inflammatory activation and microglia process elongation through Akt activation [17, 39], we performed western blot for phosphorylated NF-κB and Akt. Clozapine reduced significantly Akt phosphorylation in HT, whereas the response of HTs to NF-κB phosphorylation and of STs to both Akt and NF-κB varied (Supplementary Fig. 11a, b). Sulforaphane especially induces NRF2 pathway, and this pathway’s downstream targets *GCLM* and *HMOX1* were significantly upregulated on transcriptomic level in CTRL and HT untreated vs. sulforaphane as expected, but not in ST (Supplementary Fig. 11c).

ST microglia appeared less-responsive to drug treatments HT and CTRL iMGLs, so next we compared the significant DEGs between untreated and drug-treated in CTRL, ST or HT groups (*p* value < 0.05, abs log2FC > 1). Interestingly, especially ST iMGLs had heterogeneous response to drug treatments (Fig. 5c clozapine, d minocycline, e sulforaphane, Supplementary Data file 12–17). While iMGLs of all CTRL lines responded similarly to drug treatments, iMGLs of ST1 patient had stronger response to clozapine, iMGLs of ST3 and ST5 patients a stronger response to minocycline and iMGLs of ST3 patient a stronger response to sulforaphane than iMGLs of other ST patients. It is to be note that we had data from ST5 iMGL response only after minocycline treatment. The iMGLs of different HT lines showed consistent responses to both clozapine and minocycline treatments, with exception of HT5 iMGLs, which responded differently after clozapine and minocycline treatments.

Sulforaphane has been previously shown to upregulate BDNF in BV2 microglia cell line [16]. While we did not detect upregulation of *BDNF* after sulforaphane treatment in iMGLs of any individual, we noticed a significant upregulation of *BDNF* expression after clozapine and minocycline treatments in ST iMGLs (Fig. 5f). However, while minocycline has been shown to reduce expression of MHC class II genes, *TLR2* and *IL1β* in rodent microglia [13, 14, 40], minocycline treatment did not results in such gene expression changes in our iMGL cells of any individual. Instead, *HLA-DRA* expression was downregulated in CTRL but not ST or HT iMGL group after sulforaphane treatment. Similarly, *IL1β* expression was reduced only in CTRL iMGL group after clozapine and sulforaphane. None of the treatments resulted in altered *TLR2* expression in any iMGL group. Altogether, iMGLs of monozygotic twins from pairs discordant for SCZ appear to be less responsive than iMGLs of healthy controls to drug treatments that have been previously shown to affect microglia and be relevant for treating SCZ.

### ST twin iMGLs did not activate differently after LPS-induced inflammation

**Fig. 6:**
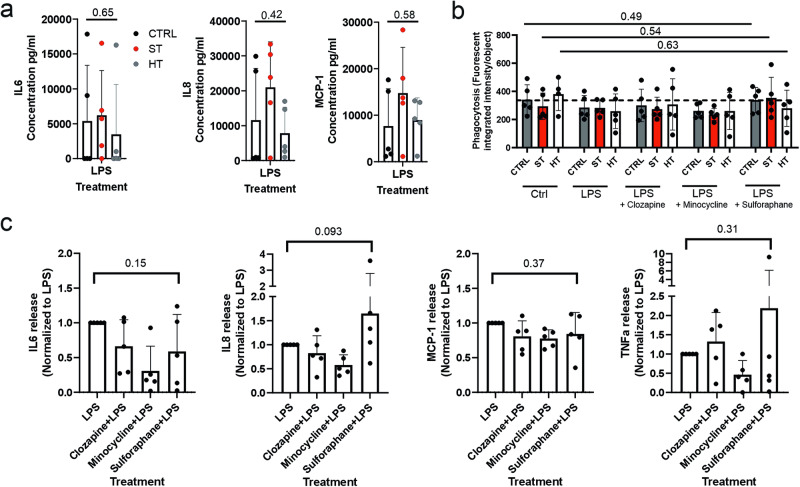
Drug treatment protection from LPS-induced inflammation in iMGLs. **a** IL6 (*H*(2) = 0.9800, *p* = 0.6500), IL8 (*H*(2) = 1.8600, *p* = 0.4163) and MCP1 (*H*(2) = 1.220, *p* = 0.5824) secretion after 24-h 100 ng/ml LPS treatment between the groups. Kruskal–Wallis test. **b** LPS effect on pHrodo-zymosan phagocytosis after 24-h LPS treatment and 6 h after adding pHrodo bioparticles (CTRL: *H*(4) = 3.397, *p* = 0.4937; ST: *H*(4) = 3.094, *p* = 0.5422; HT: *H*(4) = 2.592, *p* = 0.6282). Kruskal–Wallis test. **c** IL6 (*χ*^2^(4) = 5.400, *p* = 0.1514), IL8 (*χ*^2^(4) = 6.360, *p* = 0.0933), MCP1 (*χ*^2^(4) = 3.480, *p* = 0.3720) and TNFα (*χ*^2^(4) = 3.735, *p* = 0.3141) secretion in CTRL iMGL cultures after 30 min pre-treatment with 10 µM clozapine, 10 µM minocycline or 5 µM sulforaphane and 24-h 100 ng/ml LPS treatment. Friedman test, *n* = 5 lines.

Lipopolysaccharide (LPS) stimulation is the most common way to induce microglial inflammation. LPS activation occurs especially through TLR4-mediated NF-κB pathway activation. To see whether ST or HT iMGLs are more sensitive for LPS-induced activation, we treated iMGLs for 24 h with LPS and measured the cytokine release and phagocytosis of zymosan bioparticles. However, no differences were observed in IL6, IL8 and MCP1 secretion after LPS treatment between the groups (Fig. 6a). Similarly, LPS had no significant effect on phagocytosis in any of the groups (*p* = 0.49, CTRL; 0.54, ST; 0.63, HT; Friedman test) (Fig. 6b). Clozapine, minocycline and sulforaphane have all been reported to suppress LPS-induced activations of mouse primary microglia or BV2 microglia [17, 18, 38, 40, 41]. However, none of the drugs added 1 h prior to LPS was able to reduce IL6, IL8, MCP1 or TNFα release (Fig. 6c and Supplementary Fig. 12).

## Discussion

Microglia are brain residential immune cells which have arisen from the yolk sac and migrated to brain during development. Microglia are thus the first developed glial cell type in the brain. hiPSC differentiation allows us to generate a pure population of human microglia cell cultures [20, 42]. Human iMGLs are distinct from hematopoietic stem cells and monocytes and cluster closely with cultured human fetal and adult microglia by their RNA expression profile, as previously described [20]. We showed in this study that also iMGL gene expression was different than in hiPSC-derived macrophages. All our hiPSC-lines included in the experiment were able to differentiate into iMGLs and by their morphology and marker expression resembled resting microglia. Even though pair 4 was excluded from RNA sequencing, we were able to include all five monozygotic twins discordant for SCZ to the other experiments after preparing additional iMGL batches and careful quality controls.

hiPSC-microglia-like cells from patients with SCZ were recently shown to have increased synaptic pruning activity in neuronal co-cultures and enhanced inflammasome activity [11]. In this study, we showed that microglia from the patients with SCZ have a strong genetic component contributing to dysregulation of homeostatic and inflammatory gene expression. Since the differences between the affected twins and healthy co-twins were small, we focused on differences between twins and CTRLs. So far, human microglia are mostly studied from postmortem tissue by their morphology, cell density, and the expression of different cellular markers in particular brain areas [22, 23]. Increase of microglial inflammation markers in postmortem SCZ brains is commonly detected when studying microglia density in different brain regions, although there are contradictive results as well [6, 7, 21, 23]. HLA-DRA cell density has been found to be increased in several studies, but gene expression studies have shown no difference in patients with SCZ [21, 23, 43]. Similar variation has been seen in cytokine and chemokine expression studies [23]. However, analyses of postmortem samples represent only the late stage changes, and are affected by antipsychotic drugs while iMGLs recapitulate microglia phenotype that is only genotype-dependent.

Chronic hyperactivation leading to microglia priming has been proposed to be characterized by higher expression of inflammatory markers, higher sensitivity for activation, and exaggerated inflammatory response, when compared to unprimed microglia [44]. Although iMGLs from patients with SCZ had increased transcriptomic expression of several inflammatory markers, they showed no hyperactivation in a sense of increased cytokine production or increased migration or phagocytosis. Moreover, LPS-induced inflammation had no additional effect on iMGL proinflammatory cytokine release or phagocytosis. The latest studies have pointed out that only a subgroup of patients with SCZ show signs of increased inflammation [6]. Thus, it is possible that the patient iMGLs would not develop high inflammatory responses as hypothesized earlier.

The MHC locus is one of the earliest reported sites on the human genome associated with SCZ and the strongest association with SCZ in GWAS [9, 45]. Microglia are the main MHC class II -expressing antigen-presenting cell in the brain, which can attract antigen-specific CD4+ T lymphocytes to sites of inflammation [46, 47]. Our data suggest that upregulation of MHC class II genes is associated with clinical manifestation of SCZ. In fact, postmortem studies have found increased lymphocyte infiltration in a subset of patients with SCZ [48, 49]. Upregulation of MHC class II genes could be an early pathophysiological change that leads to SCZ manifestation later in life. According to a new hypothesis, infiltrated regulatory T lymphocytes activate astrocytes, which in turn by increasing TGFβ secretion force microglia to sustain in non-inflammatory state and promote microglial phagocytosis and pruning in patients with SCZ [50, 51]. Further studies with co-cultures, cerebral organoids and xenotransplantation of microglia [42] are needed to assess the interaction between different cell types and their effect on microglia functions.

Downregulation of microglia transcriptome in SCZ have been shown by studies integrating RNA-sequence and genetic data [52]. Downregulation of extracellular matrix constitution genes was detected from both twins compared to healthy individuals. Alterations in hepatic fibrosis and GP6 signaling pathways in microglia from patients with SCZ are shared with our previous studies in hiPSC-cortical neurons and hiPSC-astrocytes of the same individuals [35, 36] indicating universal dysregulation of these pathways. The hepatic fibrosis pathways consists of mostly different collagen genes, which are not liver-specific despite the name of the pathway. Extracellular matrix molecules have multiple roles during development to support neuronal migration and synapse formation [53]. Extracellular matrix components may induce or suppress microglial activation, their proliferation, migration and production of inflammatory cytokines as their ability to regulate synaptogenesis and neuronal transmission [53–55].

Neurogenesis is strongly influenced by microglia in the production, maturation, and integration of new neurons into circuitry by inducing neurotrophic factors and pruning weak synapses [27]. In addition, microglia-derived pro-inflammatory molecules have been shown to inhibit neurogenesis [56, 57]. We observed reduced expression of neurotrophic factors and transmitter receptors in iMGLs of affected twin pairs indicating microglia-regulated changes in modulation of neural maturation and neural transmission in patients with SCZ, while the effect on neuronal activity in co-culture system showed only subtle changes in the presence of SCZ iMGLs.

In summary, these data suggest that the increased risk to develop SCZ is associated with cell-autonomous downregulation of extracellular matrix and growth factor binding related transcriptional changes, and upregulation of inflammatory genes with clinical illness in microglia, without increased microglial pro-inflammatory activation. Microglia responses to clozapine, minocycline and sulforaphane differed from each other especially for individual patients in the group of SCZ. These findings demonstrate that drug candidates for treatment of SCZ and possibly for other mental disorders need to be tested in patient-derived models and not only in material derived from animals or healthy individuals.

### Limitation of the study

Microglia adapt rapidly to environment changes and ex vivo microglia change their expression profile compared to in vivo [58]. Thus, cultured microglia may not capture all gene expression changes, but human microglia states are conserved across different in vivo and hiPSC-microglia experimental models although some key homeostatic genes have lower expression [59]. Also, microglia in vitro tend to be more activated and secrete more cytokines than microglia in vivo, which could mask some differences in microglial responses to inflammatory stimuli. The most common reason for a sample exclusion was spontaneous activation of microglia during differentiation, leading to cell apoptosis. This was more batch-specific phenomenon than related to disease status. As some of the observed differences between the groups were high but not significant, availability of additional twin pairs discordant for SCZ could have increased the statistical power of our findings. Additional studies with large patient cohorts are needed to further validate our findings.

## Supplementary information

Supplementary Information

Supplementary Data Files

## Data Availability

Supplementary Data files contain raw RNA seq comparisons, IPA pathway analyses, and GO terms. The RNA sequencing data will be made available in EGA (European Genome-phenome Archive) upon request to the corresponding author.
