# Supplementary material for: Genetic contribution to microglial activation in schizophrenia
Source: Mol Psychiatry. 2024 Mar 22;29(9):2622–33. doi: 10.1038/s41380-024-02529-1 (PMC11420079; doi:10.1038/s41380-024-02529-1)
Supplement: Supplementary file 1 — Supplementary Information [file 41380_2024_2529_MOESM1_ESM.docx]

**Supplementary information**

**Genetic contribution to microglial activation in schizophrenia**

Marja Koskuvi^1,2^, Elina Pörsti^1^, Tristen Hewitt1, Noora Räsänen1, Ying-Chieh Wu^1^, Kalevi Trontti^1,3,4^, Amanda McQuade^5,6,7^, Shringaa Kalyanaraman1, Ilkka Ojansuu^8^, Olli Vaurio^8^, Tyrone D. Cannon^9^, Jouko Lönnqvist^10,11^, Sebastian Therman^10^, Jaana Suvisaari^10^, Jaakko Kaprio^12^, Mathew Blurton-Jones^5,6,7^, Iiris Hovatta^1,3,4^, Markku Lähteenvuo^8^, Taisia Rolova^1^, Šárka Lehtonen^1,13^, Jari Tiihonen^1,8,14^, & Jari Koistinaho^1,15^

^1^ Neuroscience Center, University of Helsinki, Helsinki, Finland

^2^ Department of Physiology and Pharmacology, Karolinska Institutet, Stockholm, Sweden

^3^ SleepWell Research Program, Faculty of Medicine, University of Helsinki, Helsinki, Finland

^4^ Department of Psychology and Logopedics, University of Helsinki, Helsinki, Finland

^5^ Department of Neurobiology & Behavior, UC Irvine, Irvine, California, USA

^6^ Sue and Bill Gross Stem Cell Research Center, UC Irvine, Irvine, California, USA

^7^ Institute for Memory Impairments and Neurological Disorders, UC Irvine, Irvine, California, USA

^8^ Department of Forensic Psychiatry, University of Eastern Finland, Niuvanniemi Hospital, Kuopio, Finland

^9^ Department of Psychology and Psychiatry, Yale University, New Haven, Connecticut, USA

^10^ Mental Health Unit, Department of Public Health Solutions, National Institute for Health and Welfare, Helsinki, Finland

^11^ Department of Psychiatry, University of Helsinki, Helsinki, Finland

^12^ Institute for Molecular Medicine FIMM, University of Helsinki, Helsinki, Finland

^13^ A.I. Virtanen Institute for Molecular Sciences, University of Eastern Finland, Kuopio, Finland

^14^ Department of Clinical Neuroscience, Karolinska Institutet, Stockholm, Sweden

^15^Drug Research Program, Division of Pharmacology and Pharmacotherapy, University of Helsinki, FI-00014 Helsinki, Finland

**Supplementary materials and methods**

**Supplementary table 1.** Summary of patient-derived hiPSC lines used in this study.

**Supplementary table 2.** Summary of hiPSC-lines included in each experimental procedure.

**Supplementary table 3.** List of used reagents.

**Supplementary figure 1.** Expression of microglia/macrophage markers in different lines.

**Supplementary figure 2.** Characterization of iMGL cells.

**Supplementary figure 3.** Expression of inflammation-associated HLA class II genes.

**Supplementary figure 4.** Phagocytosis image analysis with pHrodo zymosan bioparticles.

**Supplementary figure 5.** Expression of complement system genes.

**Supplementary figure 6.** Immunological stainings from co-cultures.

**Supplementary figure 7.** Representative raster plots from MEA recordings.

**Supplementary figure 8.** Additional graphs from co-culture MEA recordings with and without iMGLs.

**Supplementary figure 9.** Additional MEA analysis results from ST, HT and CTRL iMGL co-cultures.

**Supplementary figure 10.** Drug treatment effect on cytokine release and phagocytosis.

**Supplementary figure 11.** NF-κB, Akt and NRF2 pathway activation in iMGLs after drug treatments.

**Supplementary figure 12.** Cytokine release after LPS in ST and HT twins.

**Supplementary materials and methods**

**Macrophage differentiation**

hiPSC-macrophages were differentiated based on the previous protocols (1,2). Briefly, hiPSCs were first differentiated to HPCs with StemDiff Hematopoetic kit similarly to microglial differentiation. HPCs were further differentiated in macrophage medium (RPMI 1640+Glutamax (Lonza) + 10% FBS + 1% Penstrep (Invitrogen)) + 100 ng/ml M-CSF (Peprotech) for two weeks on uncoated plate.

**RT-qPCR**

Around 300,000-500,000 matured microglia were collected for RNA extraction with RNeasy Mini kit (Qiagen). cDNA was synthetized using Maxima reverse transcriptase enzyme (Thermo Fisher Scientific) according to manufacturer’s instructions. Relative mRNA expression was measured using Maxima Probe qPCR Master Mix (Thermo Fisher Scientific) and Taqman primers AIF1 (Hs00610419_g1), ITGAM (Hs00167304_m1), P2RY12 (Hs00375457_m1), TREM2 (Hs00219132_m1), C1QA (Hs00381122_m1), GAS6 (Hs01090305_m1), and PROS1 (Hs00165590_m1) on Bio‐Rad CFX96 Real‐Time System (Bio‐Rad). The expression results were normalized to ACTB (Applied Biosystems, cat. 4326315E) using Q-gene program (Equation 2) (3).

**RNA sequencing**

RNA-sequencing libraries were produced with NEBNext Ultra II Directional RNA Library Prep Kit (New England Biolabs). Libraries were sequenced with NextSeq500 (Illumina). The sequencing was provided by the Biomedicum Functional Genomics Unit at the Helsinki Institute of Life Science and Biocenter Finland at the University of Helsinki. iMGL sample sequence reads were aligned to the human genome GRCh.38 using STAR aligner v2.7.2 (4) and annotated to gene exons with HTSeq v0.11.2 (5). DESeq2 (6) was used to analyze differential gene expression between ST and HT and CTRL groups.

Ingenuity Pathway Analysis (IPA) (Qiagen) was used for pathway enrichment analyses. Absolute fold change >2 and false discovery rate <0.05 was applied as a cut-off for the differentially expressed genes in the analysis. The analysis was performed with additional filters for species, tissue and cells (human, nervous system and CNS lines & Immune cells & other cells). GO enrichment analysis was done with Gprofiler2 package (7). Upregulated and downregulated genes that passed cut-off of absolute fold change >2 and false discovery rate <0.05 were ordered by fold change, and GO enrichment analysis was performed using the gost() function in gprofiler2_0.2.1 with the following parameters: organism = ‘hsapiens’, ordered_query = TRUE, user_threshold = 0.05, correction_method = ‘gSCS’ and sources = c(‘GO:BP’,‘GO:MF’,‘GO:CC’,’). For representative GO plots the term size was restricted to 1,000 and the top ten terms by −log10(p-value) were plotted with exclusion of successive terms containing identical evidence codes. Cut-off values: absolute fold change >2 and nominal p-value<0.05 were used. 10 DEGs with the highest and lowest fold changes were taken, excluding genes, in which expression was zero as untreated.

**Immunocytochemistry**

Coverslips (9 mm, circular) were pre-coated with Poly-L-ornithine (Sigma) and dried on 48-well plate. Around 10,000-20,000 cells in 80 µl of 1:6 Matrigel (Corning) in iMGL differentiation medium was seeded on top of the coverslip. The Matrigel bubble was let harden overnight in +37 °C 5 % CO_2_ incubator and iMGL- maturation medium was added on the next day for two days. The cells were fixed with 4 % PFA for 20 mins. The samples were blocked with 0.1% BSA for 1 hours and the following antibodies were used: goat-α-IBA1 (Novus Biologicals) 1:500, rabbit-α-CX3CR1 (Thermo Fisher) 1:200, goat-α-TREM2 (R&D Systems) 1:200, and rabbit-α-P2RY12 (Sigma) 1:1000 overnight. The next day, secondary antibodies chicken-α-goat 488 (Invitrogen) and chicken-α-rabbit 647 (Invitrogen) 1:1000 were added for 1 hour. Nuclei were stained with DAPI. Images were taken with ZEISS Axio Imager M1 microscope.

**Phagocytosis**

For phagocytosis assay 20,000 cells/well were plated onto a 96-well plate for two-day maturation in iMGL maturation medium and treatments added for 24 h. 10 µg of pHrodo zymosan A BioParticles (Invitrogen) were added before live imaging with IncuCyte S3 (Sartorius). Images were taken each 30 min with 20x objective for 6 hours. Analysis was done using IncuCyte analysis software (2018B). Red or Green integrated intensity was normalized to Red or Green object count at each data point, respectively. Cells without BioParticles or with BioParticles and latrunculin B (0.5 µg/ml for 30 min pre-treatment) were used for background extraction. The experiment was repeated 2-3 times with 2-3 replicated wells for each line.

**Migration**

Cell migration was assessed with transwell system. Either iMGL-medium only, iMGL-medium with 100 µM ADP (Sigma; cat. A2754) or 100 µM ATP (Sigma; cat. A2383) was added onto a 24-well plate. Totally 20,000 differentiated microglia were seeded into 3 µm pore size transwell inserts (Sarstedt) for 4 hours. The bottoms of the 24-well plate wells were imaged with IncuCyte S3. IncuCyte analysis software (2018B) was used to calculate the number of migrated cells per mm^2^ (phase object count). The experiment was repeated 2-3 times/line with 2-3 replicates.

**MTT (Viability) assay**

20,000 cells was plated into 96-well plate and matured two days in iMGL-maturation medium. The drug treatments were added on third day for 24 hours in iMGL-maturation medium. 0.5 mg/ml MTT was added into the wells and incubated for three hours. After three hours, the medium was replaced with DMSO and the plate was incubated overnight. The absorbance at 585 was measured in duplicates with ThermoFisher Multiskan FC plate reader. The drug concentrations were tested with 4 independent experiments with two CTRL, two HT and two ST lines (n=6 lines) with 2-3 replicates.

**Co-culture**

HPCs were differentiated for 20 days in iMGL differentiation medium. Fourteen days before seeding, hiPSCs derived from a control individual (SZ18) carrying a doxycycline (Dox) inducible NGN2 expression vector were differentiated into cortical neurons as previously described (8). In short, hiPSCs containing NGN2 gene were incubated in 2 µg/ml Dox (Biogems) for 24 hours. The medium was then changed to N2 medium (DMEM/F12, 1X GlutaMAX, 1X N2, and 0.3% glucose) supplemented with 10 µM SB431542 (Sigma), 0.1 µM LDN193189 (Sigma), 2 µM XAV939 (Biogems), and 2 µg/ml Dox. After 24 hours, cells expressing NGN2 were selected for with 5 µg/ml puromycin (MP Biomedicals) and the supplements (except for Dox) were reduced to half their normal concentrations. The selection was removed 24 hours later, and the cells incubated in standard N2 medium plus supplements. On day 4 of differentiation, the NPCs were detached with Accutase (StemCell Technologies) and seeded at 70 000 cells/well in a 1:1 ratio with rat astrocytes dissected from the cortex of E18 rat embryos onto 24-well multi-electrode array (MEA; Axion Biosystems) or onto standard Matrigel-coated 48-well plates with coverslips. The MEA plate had been coated with 50 µl 0.5 mg/ml poly-ornithine 1 day prior, followed by seeding with 10 µg/ml laminin to ensure adequate adherence to the electrodes. The neuron-astrocyte co-culture was maintained in NBM+ medium (Neurobasal medium, 1X GlutaMax, 0.5X MEM NEAA, and 0.3% glucose) supplemented with 1:50 B27 without vitamin A, 10 ng/ml BDNF (PeproTech), 10 ng/ml GDNF (PeproTech), 10ng/ml CNTF (PeproTech) and 2 µg/ml Dox for 7 days. Three days after seeding, 10 µM FUDR (Tocris) was added for 24 hours to eliminate any proliferating cells still in the culture. Half media changes were done every 2-3 days. Seven days after seeding, iMGLs (now differentiated for 20 days in iMGL differentiation medium) were scraped from the bottom of the plate and seeded at a 1:1:6 neuron:astrocyte:iMGL ratio. The culture medium was then simplified to NBM+ medium supplemented with 1:50 B27 without vitamin A and 50 ng/ml IL-34. Half media changes were conducted every 2-3 days.

**MEA recoding**

Spontaneous neuronal activity was recorded 3 times a week with the Axion Maestro Edge system with environmental controls set at 5% CO2 and 37°C. Spike sorting was conducted using the Axion-provided AxlS Navigator software and spike threshold was set to 5x standard deviation of estimated noise. Wells were excluded if the number of active electrodes was <30% (5/16), where an active electrode recorded at least 5 spikes per minute. Burst detection was conducted with the Axion-provided Neural Metric Tool with the Inter-spike Interval Threshold algorithm and default parameters: a minimum of 5 spikes needed to occur per burst, a maximum of 100 ms for inter-spike interval within a burst, and a minimum of 50 spikes within 100 ms with 35% electrode coverage were needed for bursts to be considered a network burst. The experiment was done with three twin pairs and three healthy controls (all females). Due to the several repetitive timepoints and expected variation in activity, MEA recordings were performed with several replicates (n=6), which then reduced the feasible number of patient lines included in the recordings.

**CBA**

Totally 20,000 differentiated microglia/well was seeded into 96-well plate in iMGL maturation medium for two days. The following treatments were added for 24 hours: 20 ng/ml IL1β, 20 ng/ml TNFα, 20 ng/ml IFNγ (all from Peprotech), 100 ng/ml LPS (Sigma) or drug treatments as mentioned before. CBA for Human IL6, Human IL8, Human TNF, and Human MCP-1 (all from BD Biosciences) was performed according the manufactor’s instructions with BD Accuri flow cytometry.

**Western blot**

Cells were lysed in RIPA Lysis Buffer containing protease and phosphatase inhibitors (all from Thermo Fisher Scientific) and denaturated. Total protein (10 μg) was loaded and separated on a 4-20% Mini-PROTEAN® TGX™ Gels (Bio-Rad) and then transferred to polyvinylidene fluoride (PVDF) membranes (Bio-Rad) using Trans-Blot Turbo Transfer System (Bio-Rad). The membranes were blocked at room temperature for 1 h in 5% fat-free milk in TBST buffer, after which the membranes were incubated at 4°C overnight with the primary antibody diluted in 5% BSA – 0.02% Na azide in TBST buffer. The primary antibody used were rabbit α-Phospho-Akt (Cell Signaling, 1:1000), rabbit α-Akt (Cell Signaling, 1:1000), rabbit α-Phospho-NF-κB p65 (Cell Signaling, 1:1000), rabbit α-NF-κB p65 (Cell Signaling, 1:1000). The membranes were incubated with the secondary antibody diluted in 5% fat-free milk in TBST buffer at room temperature for 1 h. The secondary antibodies used was HRP-conjugated goat anti-rabbit IgG (Invitrogen, 1:40 000). The bands were visualized by ECL or ECL Plus Western Blotting Substrate (Thermo Fisher Scientific, cat. 32106 and cat. 32132), and the images were processed and acquired using G:BOX Chemi XX6 system (Syngene). The intensity of the bands was quantified using ImageJ software.

**Statistics**

As sample size was mostly four or five patients in a group, only non-parametric test were used. No sample size estimate was calculated. All statistical analyses of qPCR data, CBA and functional assays were performed using Prism (version 8.4.2 or 9.4.1; GraphPad Software, La Jolla, California), with statistical significance set at p < 0.05. Transcriptomic data were analyzed as described above, using FDR correction set at a 5% threshold (q < 0.05). Only genes that passed this FDR threshold were considered as significant DEGs. For comparing treatment effects paired tests were used: Friedman test and Wilcoxon matched-pairs signed rank test. For group comparison Kruskal-Wallis test and Mann-Whitney test were used. Test were two-tailed. Exact p-values are given in the figures. Grubbs' test for outlier detection was used. Each data point represents separate patient line with pooled replicates. The data are presented as Mean +/- SD, except for phagocytosis curves as Mean +/- SEM.

In MEA studies when the data did not contain missing values, an RM 2-way ANOVA with the Geisser-Greenhouse correction was used. Sphericity was not assumed and data were matched by time point. Simple effects within each separate time point was used as a readout for multiple comparisons using Tukey’s post-hoc test or simple effects within group compared to week 4 time point using Dunnett's multiple comparisons test (α=0.05). If values were missing from the data (due to inadequate signal or electrode coverage), a mixed-effects model with the Geisser-Greenhouse correction was used followed by Tukey’s multiple comparisons tests.

**Supplementary references**

1. Mukherjee C, Hale C, Mukhopadhyay S. A Simple Multistep Protocol for Differentiating Human Induced Pluripotent Stem Cells into Functional Macrophages. In: Rousselet G, editor. Macrophages [Internet]. New York, NY: Springer New York; 2018 [cited 2022 Jun 7]. p. 13–28. (Methods in Molecular Biology; vol. 1784). Available from: http://link.springer.com/10.1007/978-1-4939-7837-3_2

2. Shi J, Xue C, Liu W, Zhang H. Differentiation of Human‐Induced Pluripotent Stem Cells to Macrophages for Disease Modeling and Functional Genomics. Curr Protoc Stem Cell Biol. 2018 Dec 10;e74.

3. Muller PY, Janovjak H, Miserez AR, Dobbie Z. Processing of gene expression data generated by quantitative real-time RT-PCR. BioTechniques. 2002 Jun;32(6):1372–4, 1376, 1378–9.

4. Dobin A, Davis CA, Schlesinger F, Drenkow J, Zaleski C, Jha S, et al. STAR: ultrafast universal RNA-seq aligner. Bioinformatics. 2013 Jan;29(1):15–21.

5. Anders S, Pyl PT, Huber W. HTSeq--a Python framework to work with high-throughput sequencing data. Bioinformatics. 2015 Jan 15;31(2):166–9.

6. Love MI, Huber W, Anders S. Moderated estimation of fold change and dispersion for RNA-seq data with DESeq2. Genome Biol. 2014 Dec;15(12):550.

7. Kolberg L, Raudvere U, Kuzmin I, Vilo J, Peterson H. gprofiler2 -- an R package for gene list functional enrichment analysis and namespace conversion toolset g:Profiler. F1000Research. 2020 Nov 17;9:709.

8. Nehme R, Zuccaro E, Dia Ghosh S. Combining NGN2 Programming with Developmental Patterning Generates Human Excitatory Neurons with NMDAR-Mediated Synaptic Transmission. Cell Rep. 2018 May 22;23(8):2509-2523.

**Supplementary tables**

**Supplementary table 1. Summary of hiPSC lines used in this study.** Pair 2 has been excluded previously from the cohort in Tiihonen, et al. 2019 (as the affected one did not fullfil PANSS criteria) and one control line (SZ14) was not used in this study. PANSS = Positive and Negative Syndrome Scale

| **Abbreviations of hiPSC lines** | **Group** | **Age at**  **biopsy**  **(years)** | **Sex** | **Medication** | **Age at symptoms appeared (years)** | **PANSS total** |
| --- | --- | --- | --- | --- | --- | --- |
| SZ1 (HT1) | Healthy twin | 47 | F | - |  | 49 |
| SZ2 (ST1) | Affected twin | 47 | F | Clozapine | 16 | 113 |
| SZ5 (ST3) | Affected twin | 66 | M | Zuclopenthixol | 17 | 77 |
| SZ6 (HT3) | Healthy twin | 66 | M | - |  | 31 |
| SZ7 (ST4) | Affected twin | 69 | F | Previously clozapine, now sertindole and quetiapine | 17 | 99 |
| SZ8 (HT4) | Healthy twin | 69 | F | - |  | 35 |
| SZ9 (HT5) | Healthy twin | 45 | F | - |  | 31 |
| SZ10 (ST5) | Affected twin | 45 | F | Clozapine | 32 | 53 |
| SZ11 (CTRL1) | Healthy individual | 44 | M | - |  | 30 |
| SZ12 (CTRL2) | Healthy individual | 59 | F | - |  | 30 |
| SZ13 (CTRL3) | Healthy individual | 49 | F | - |  | 30 |
| SZ15 (ST6) | Affected twin | 40 | M | Olanzapine and quetiapine | 20 | 57 |
| SZ16 (HT6) | Healthy twin | 40 | M | - |  | 30 |
| SZ17 (CTRL5) | Healthy individual | 63 | M | - |  | 30 |
| SZ18 (CTRL6) | Healthy individual | 50 | F | - |  | 30 |

**Supplementary table 2. Summary of hiPSC-lines included in each experimental procedure.**

|  | **SZ1** | **SZ2** | **SZ5** | **SZ6** | **SZ7** | **SZ8** | **SZ9** | **SZ10** | **SZ11** | **SZ12** | **SZ13** | **SZ15** | **SZ16** | **SZ17** | **SZ18** |
| --- | --- | --- | --- | --- | --- | --- | --- | --- | --- | --- | --- | --- | --- | --- | --- |
| **RNAseq** | x | x | x | x |  |  | x | x | x | x |  | x | x | x | x |
| **qPCR** |  |  |  |  |  |  |  |  | x | x | x |  |  | x | x |
| **ICC** | x | x | x | x | x | x | x | x | x | x | x | x | x | x | x |
| **Phagocytosis** | x | x | x | x | x | x | x | x | x | x | x | x | x | x | x |
| **MTT** | x |  | x |  |  |  |  |  |  |  | x | x | x | x |  |
| **CBA** | x | x | x | x | x | x | x | x | x | x | x | x | x | x | x |
| **co-culture microglia** | x | x |  |  | x | x | x | x |  | x | x |  |  |  | x |
| **Co-culture NGN2-neuron** |  |  |  |  |  |  |  |  |  |  |  |  |  |  | x |
| **Macrophage diff** |  |  |  |  |  |  |  |  | x |  | x |  |  | x |  |
| **Migration** | x | x | x | x | x | x | x | x | x | x | x | x | x | x |  |
| **WB** | x | x | x | x |  |  | x | x |  |  |  | x | x |  |  |

**Supplementary table 3. List of used reagents.**

| **Antibodies** | **Producer** | **Catalog number** |
| --- | --- | --- |
| goat-α-IBA1 | Novus Biologicals | NB100-1028 |
| rabbit-α-CX3CR1 | Thermo Fisher Scientific | PA5-19910 |
| goat-α-TREM2 | R&D Systems | AF1828 |
| rabbit-α-P2RY12 | Sigma | HPA014518 |
| rabbit α-Phospho-Akt | Cell Signaling | 4060 |
| rabbit α-Akt | Cell Signaling | 4691 |
| rabbit α-Phospho-NF-κB p65 | Cell Signaling | 3033 |
| rabbit α-NF-κB p65 | Cell Signaling | 8242 |
| chicken-α-goat 488 | Invitrogen | A21467 |
| chicken-α-rabbit 647 | Invitrogen | A21443 |
| HRP-conjugated goat anti-rabbit IgG | Invitrogen | A16096 |
| **Cell culture reagents** | **Producer** | **Catalog number** |
| E8 medium | Gibco | A1517001 |
| Matrigel | Corning | 356231 |
| EDTA | Invitrogen | 15575-038 |
| ReLeSR | StemCell Technologies | 5872 |
| DMEM/F12 | Gibco | 21331020 |
| ITS-G | Invitrogen | 41400045 |
| B27 supplement | Invitrogen | 17504044 |
| Glutamax | Invitrogen | 35050-038 |
| NEAA | Invitrogen | 11140050 |
| N2 supplement | Invitrogen | 17502-048 |
| Penstrep | Invitrogen | 15140-122 |
| Insulin | Sigma | 19278 |
| Monothioglycerol | Sigma | M1753 |
| RPMI 1640 | Lonza | BE12-167 |
| LPS | Sigma | 2630 |
| Poly-L-ornithine | Sigma | P4957 |
| **Small molecules, cytokines, and growth factors** | **Producer** | **Catalog number** |
| Y-27632 | Sigma | Y0503 |
| IL34 | PeproTech | 200-34 |
| TGFβ-1 | PeproTech | 100-21 |
| M-CSF | PeproTech | 300-25 |
| CX3CL1 | PeproTech | 300-31 |
| CD200 | Biolegend | 77002 |
| Doxycycline | Biogems | 2431450 |
| SB431542 | Sigma | S4317 |
| LDN193189 | Sigma | SML0559 |
| XAV939 | Biogems | 2848932 |
| BDNF | PeproTech | 450-02 |
| GDNF | PeproTech | 450-10 |
| CNTF | PeproTech | 450-13 |
| Puromycin | MP Biomedicals | 100552 |
| FUDR | Tocris | 50-91-9 |
| Clozapine | Sigma | C-6305 |
| Minocycline | Sigma | M9511 |
| D,L-Sulforaphane | Santa Cruz | SC-207495 |
| IL1β | PeproTech | 200-01B |
| TNFα | PeproTech | 300-01A |
| IFNγ | PeproTech | 300-02 |
| **Primers** | **Producer** | **Catalog number** |
| AIF1 | Thermo Fisher Scientific | Hs00610419_g1 |
| ITGAM | Thermo Fisher Scientific | Hs00167304_m1 |
| P2RY12 | Thermo Fisher Scientific | Hs00375457_m1 |
| TREM2 | Thermo Fisher Scientific | Hs00219132_m1 |
| C1QA | Thermo Fisher Scientific | Hs00381122_m1 |
| GAS6 | Thermo Fisher Scientific | Hs01090305_m1 |
| PROS1 | Thermo Fisher Scientific | Hs00165590_m1 |
| ACTB | Applied Biosystems | 4326315E |
| **Kits** | **Producer** | **Catalog number** |
| StemDiff Hematopoetic kit | StemCell Technologies | 05310 |
| RNeasy Mini kit | Qiagen | 74106 |
| NEBNext Ultra II Directional RNA Library Prep Kit | New England Biolabs | E7760 |
| **Assay regents** | **Producer** | **Catalog number** |
| pHrodo zymosan A BioParticles | Invitrogen | P35364 and P35365 |
| ADP | Sigma | A2754 |
| ATP | Sigma | A2383 |
| Transwell inserts | Sarstedt | 83.3932.300 |
| Human IL6 Flex set (CBA) | BD Biosciences | 558276 |
| Human IL8 Flex set (CBA) | BD Biosciences | 558277 |
| Human TNF Flex set (CBA) | BD Biosciences | 560112 |
| Human MCP-1 Flex set (CBA) | BD Biosciences | 558287 |
| Mini-PROTEAN® TGX™ Gels | Bio-Rad | 4561094 |
| ECL (Plus) Western Blotting Substrate | Thermo Fisher Scientific | 32106 and 32132 |
| **Main software** | **Version** | **Producer** |
| STAR aligner | v2.7.2 | Dobin, et al. 2013 |
| HTSeq | v0.11.2 | Anders, et al. 2015 |
| DESeq2 |  | Love, et al. 2014 |
| Ingenuity Pathway Analysis |  | Qiagen |
| Gprofiler2 |  | Kolberg, et al. 2020 |
| GraphPad Prism | v8.4.2 or v9.4.1 | GraphPad |


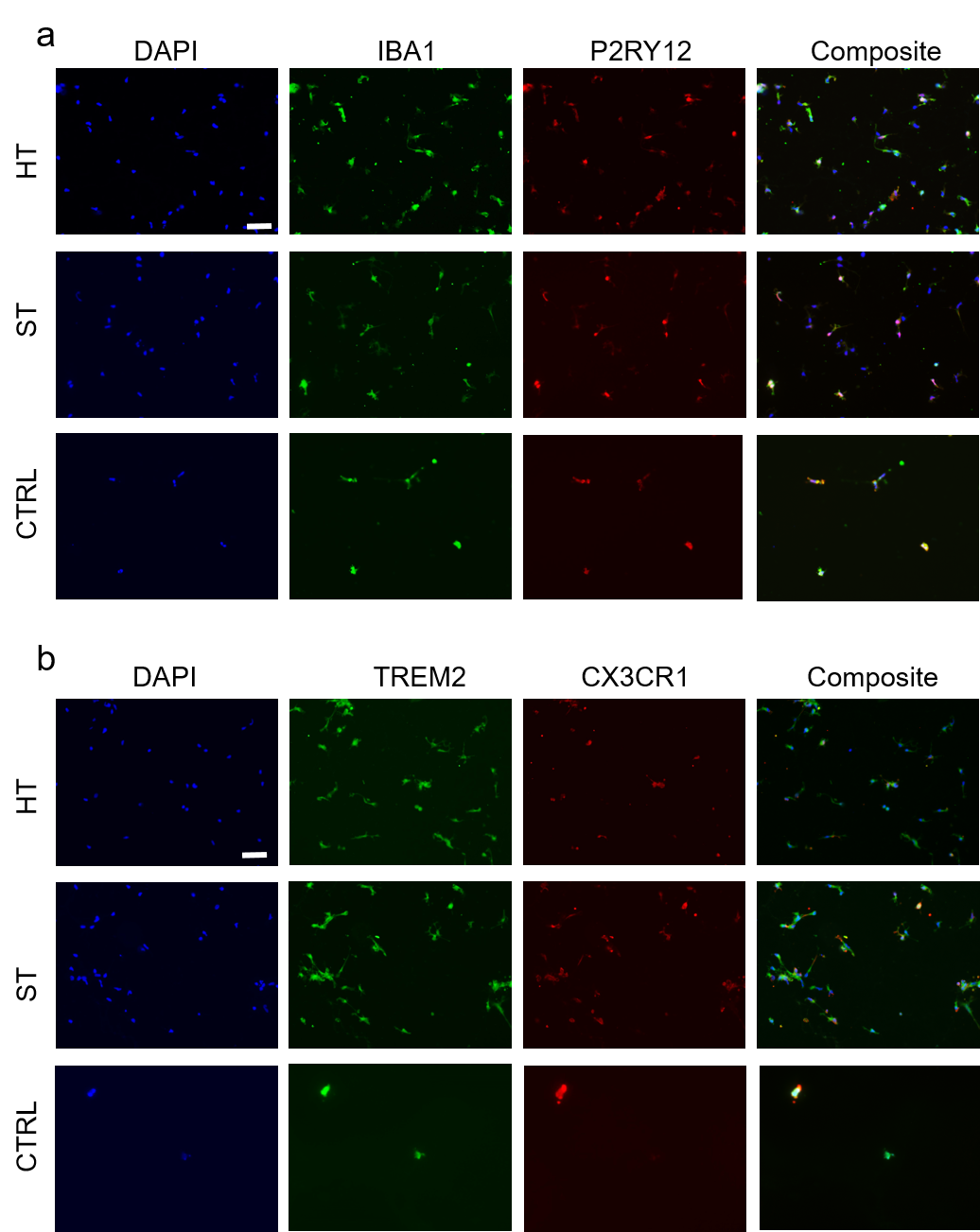
**Supplementary figures**

**Supplementary figure 1. Expression of microglia/macrophage markers in different lines.** Related to Figure 1b. Expression of **a)** pan-macrophage marker IBA1, and microglia-specific markers P2Y12, **b)** TREM2 and CX3CR1 in CTRL, ST and HT lines. Nuclei stained with DAPI. Scale bar 50 µm.


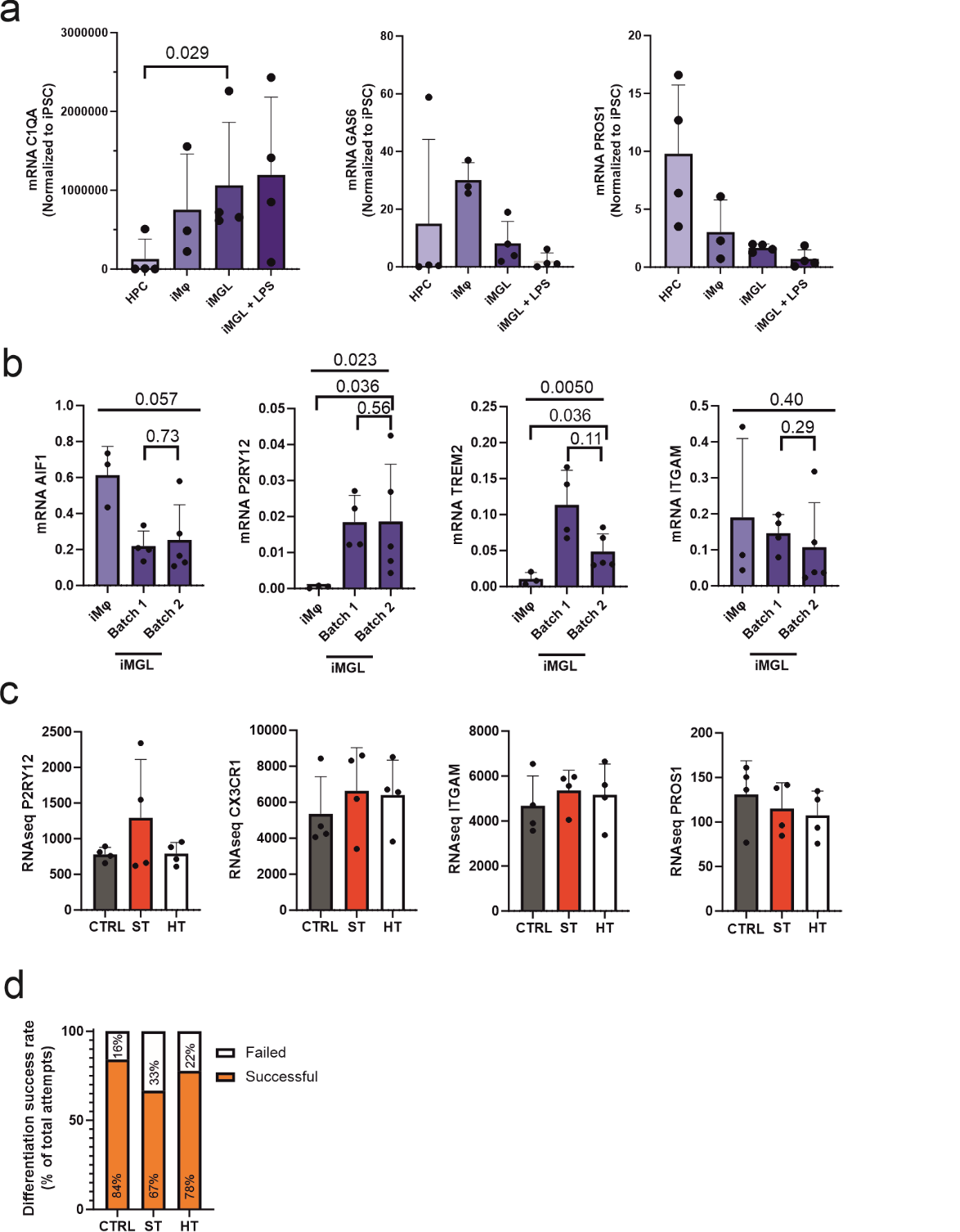


**Supplementary figure 2.** Characterization of iMGL cells**.** Related to Figure 1c. **a)** mRNA expression of *C1QA* (HPC vs. iMGL U=0, p=0.0286), *GAS6,* and *PROS1*. **b)** Comparison of two separate iMGL batches (iMφ CTRLs n=3, iMGL CTRLs n=4) RNA expression of AIF1 (H(2)=5.426, p=0.0566; U=8, p=0.7302), P2RY12 (H(2)=6.542, p=0.0233; U=7, p=0.5556; U=0, p=0.0357), TREM2 (H(2)=7.923, p=0.0050; U=0, p=0.0357; U=3, p=0.1111), and ITGAM (H(2)=1.964, p=0.3999). Kruskal-Wallis test and Mann-Whitney test, n=3-4 lines. **c)** Gene expression between the groups in homeostatic genes *P2RY12*, *CX3CR1, ITGAM,* and *PROS1*. RNAseq n=4 cell lines per group. **d)** Differentiation success rate from total attempts. The differentiation was considered successful if the cells were used for experiments (n=18-19, χ2(2)=1.61, p=0.448, n.s.).


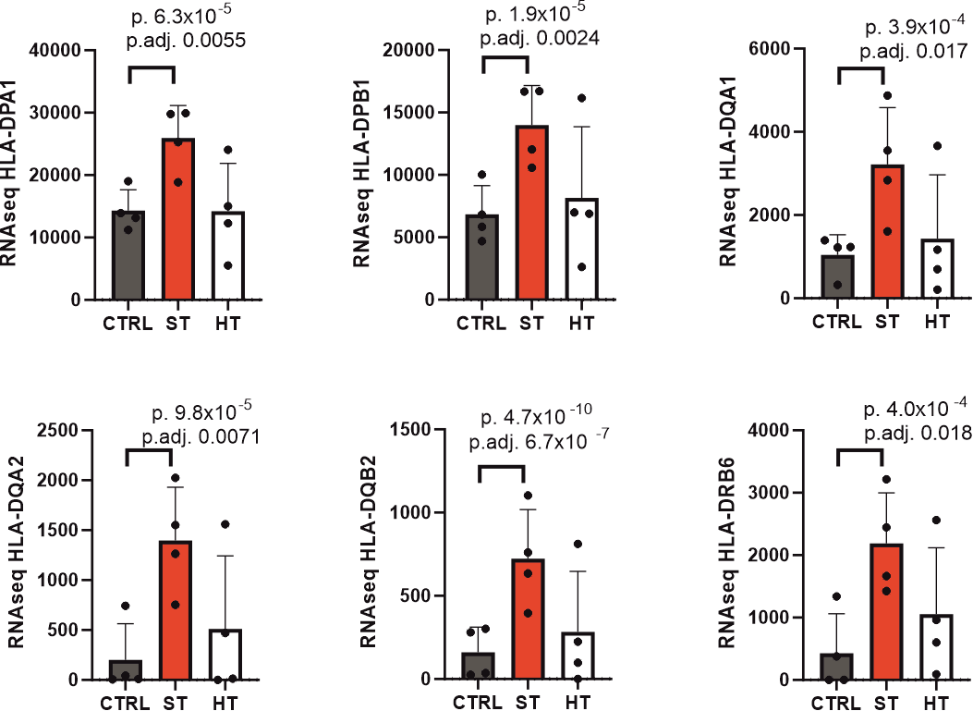


**Supplementary figure 3.** **Expression of inflammation-associated HLA class II genes.** Related to Figure 2a. Gene expression of different HLA genes: *HLA-DPA1, HLA-DPB1, HLA-DQA1, HLA-DQA2, HLA-DQB2*, and *HLA-DRB6*. RNAseq n=4 cell lines per group.


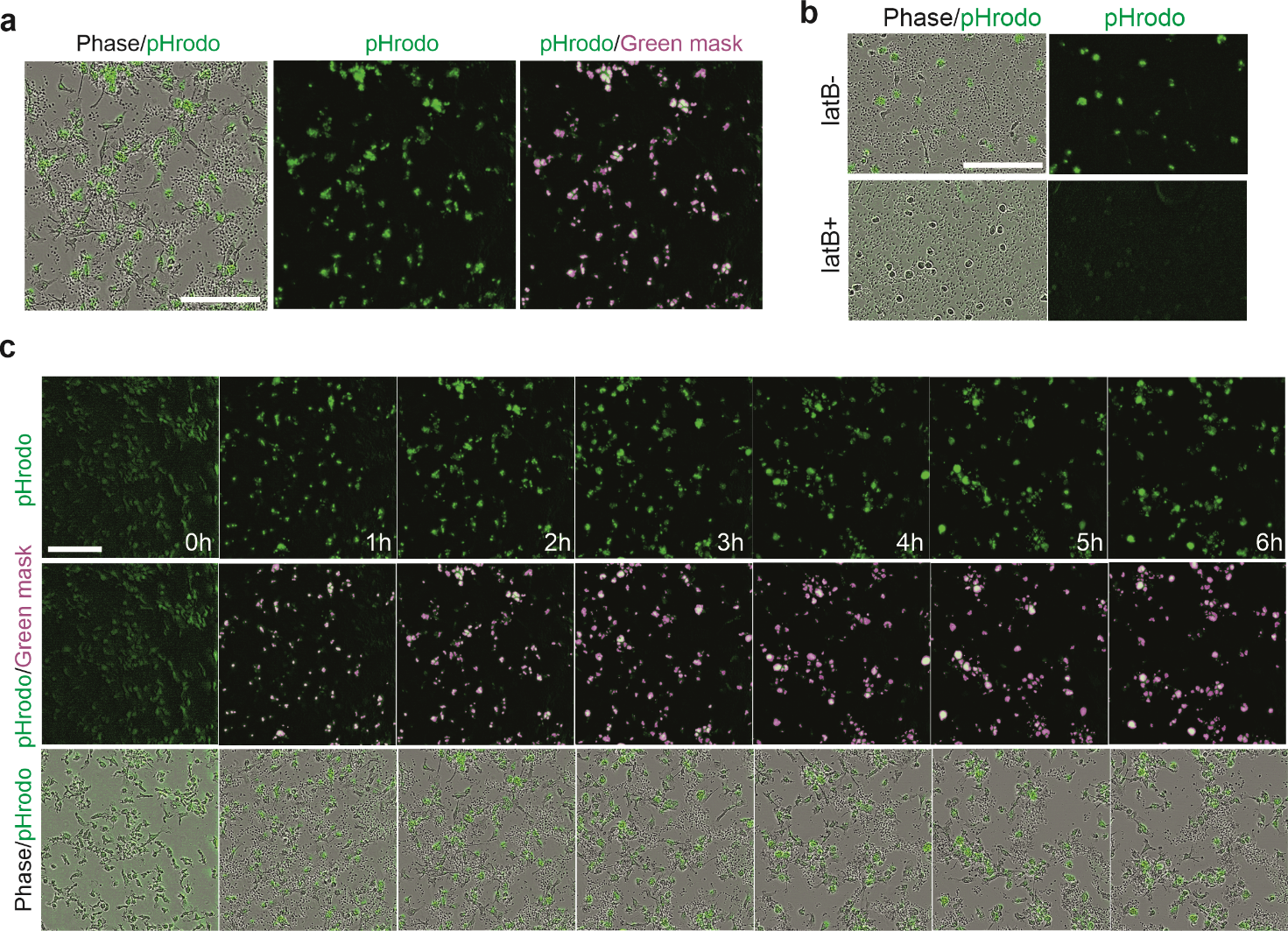


**Supplementary figure 4. Phagocytosis image analysis with pHrodo zymosan bioparticles.** Related to Figure 2g-h. **a)** Representative images from phase contrast/pHrodo (green), pHrodo and green mask (magenta) used for analysis. **b)** Phase/pHrodo and pHrodo (green) from wells with and without latrunculin B (latB). **c)** Representative images from 6-hour time-lapse imaging. Scale bar 100 µm in all.


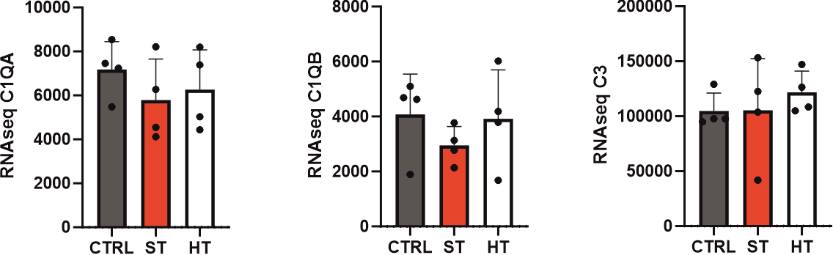


**Supplementary figure 5.** **Expression of complement system genes.** Gene expression of *C1QA*, *C1QB* and *C3* in iMGLs by groups based on RNAseq. RNAseq n=4 cell lines per group.


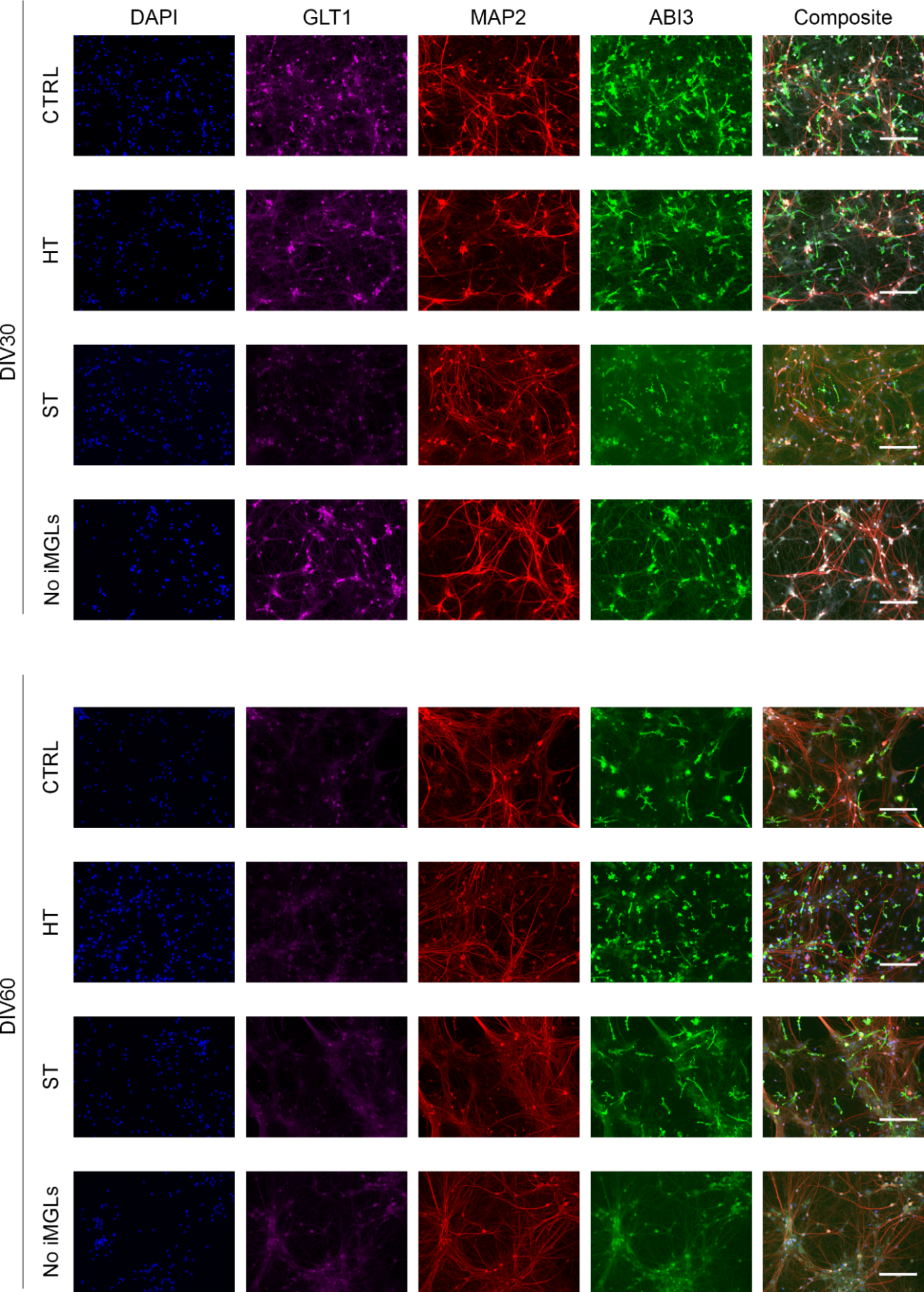


**Supplementary figure 6. Immunocytochemical stainings from co-cultures.** Related to Figure 3d. Co-cultures with NGN2-neurons (MAP2, red), rat astrocytes (GLT1, magenta) and with/without iMGLs (ABI3, green) were stained after 30 or 60 days in vitro (DIV). Nuclei were stained with DAPI (blue). Scale bars 150 µm.


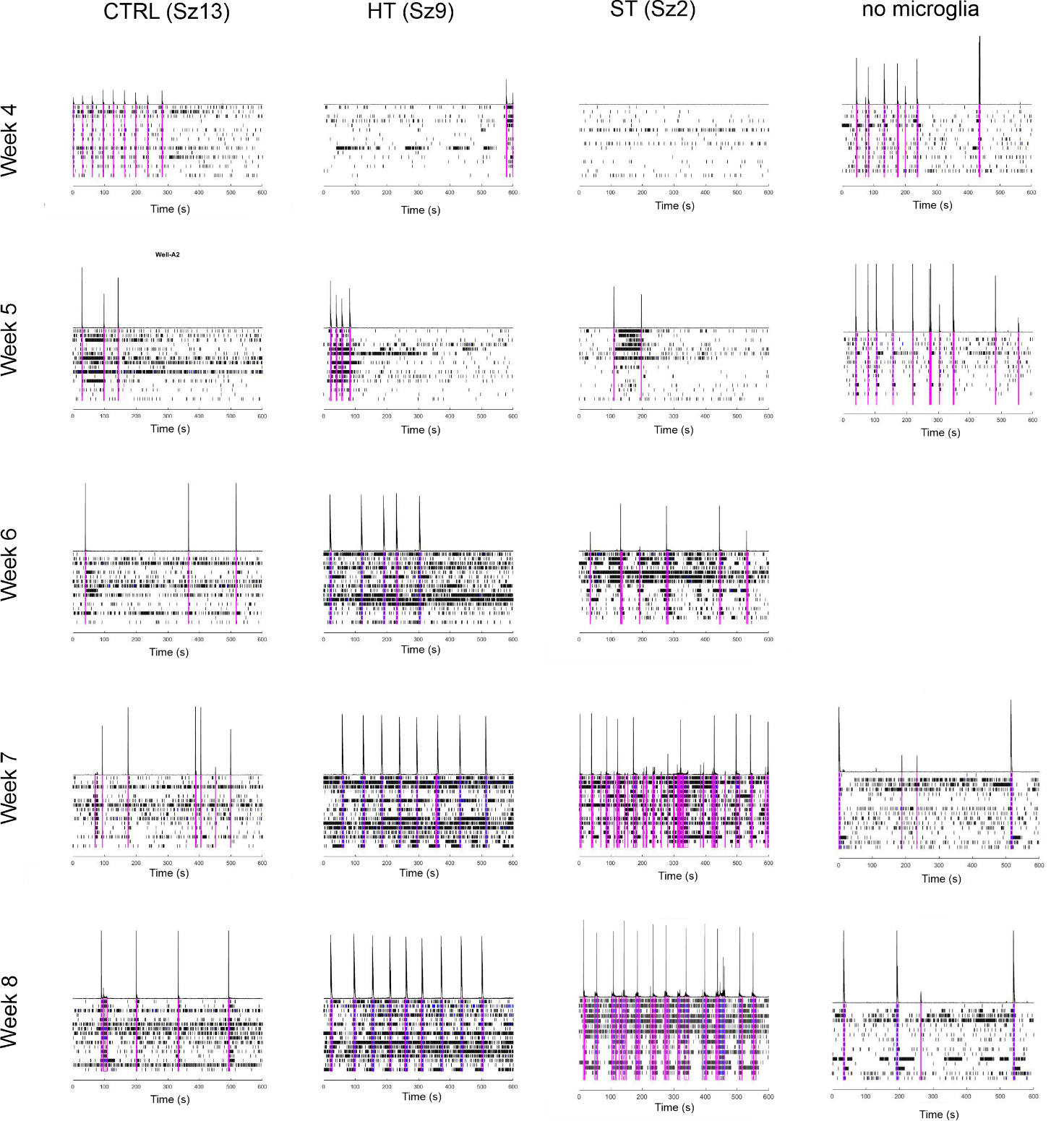


**Supplementary figure 7. Representative** **raster plots from MEA recordings.** Related to Figure 3e. Raster plots showing activity from 16 electrodes in 600 seconds from co-cultures with CTRL, HT and ST iMGLs and without iMGLs from the same well during weekly recordings. Spikes detected on individual electrodes marked with black lines, bursts with blue lines and network bursts with purple boxes. No data recorded from no-microglia well at 6 weeks.


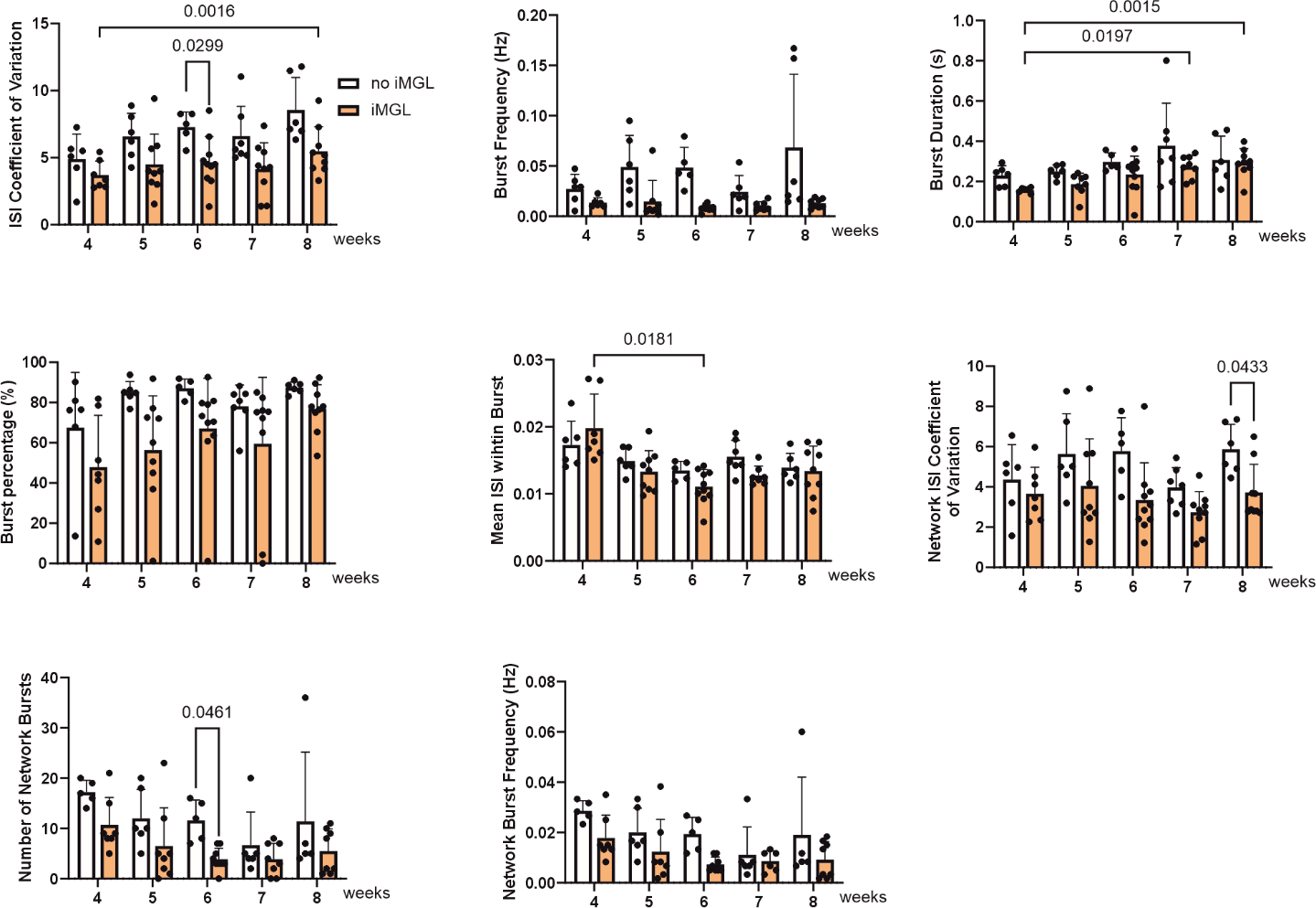


**Supplementary figure 8. Additional graphs from co-culture MEA recordings with and without iMGLs.** Related to Figure 3f. The results are presented as ISI coefficient of variation (95% CI of diff.= -5.021 to -0.2202, adj.p=0.0299; 95% CI of diff.= -2.663 to -0.9038, adj.p=0.0016), Burst frequency, Burst duration (95% CI of diff.= -0.2072 to -0.02395, adj.p= 0.0197; 95% CI of diff.= -0.1955 to -0.07508, adj.p=0.0015), Burst percentage, Mean ISI within burst (95% CI of diff.= 0.001779 to 0.01569, adj.p=0.0181), Network ISI coefficient of variation (95% CI of diff.= -4.255 to -0.05488, adj.p=0.0433), Number of network burst (95% CI of diff.= -15.27 to -0.1542, adj.p=0.0461), and Network burst frequency. Wells were excluded if the number of active electrodes was <30%. Dunnett’s (timepoints compared to week 4 timepoint) or Tukey’s (comparison between with/without iMGL groups) multiple comparisons tests were used for significance. n=5-7 wells for no iMGL and n=6-10 wells from two CTRL iMGL lines.


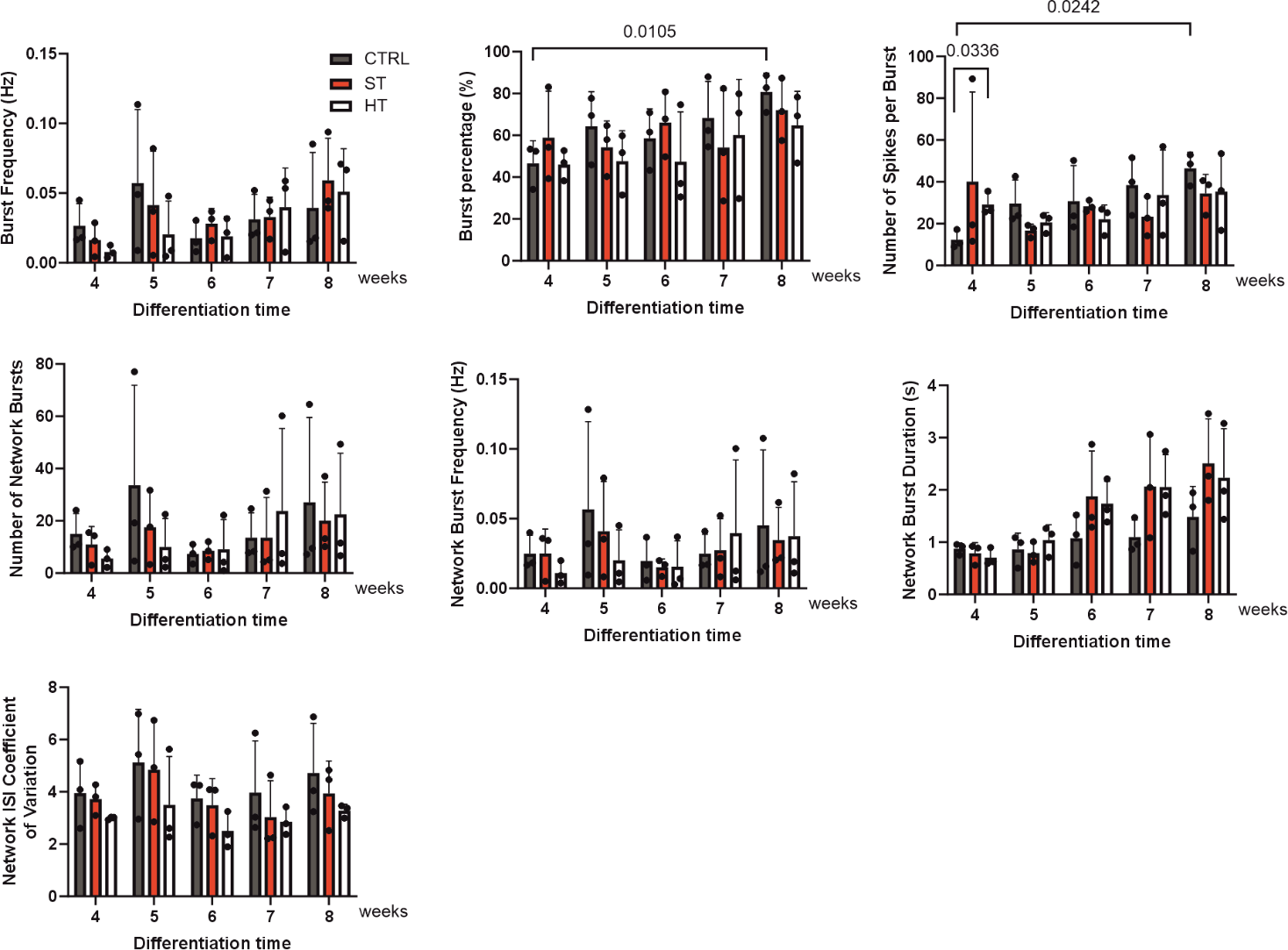


**Supplementary figure 9. Additional MEA analysis results from ST, HT and CTRL iMGL co-cultures.** Related to Figure 3g. The results are presented as Burst frequency, Burst percentage (95% CI of diff.= -49.53 to -18.78, adj.p= 0.0105), Number of spikes per burst (95% CI of diff.= -57.66 to -10.70, adj.p=0.0242; 95% CI of diff.= -31.79 to -2.044, adj.p= 0.0336), Number of network bursts, Network burst frequency, Network burst duration, and Network ISI coefficient of variation. Dunnett’s (timepoints compared to week 4 timepoint) or Tukey’s (comparison between with/without iMGL groups) multiple comparisons tests were used for significance. n= 3 lines per group.

**
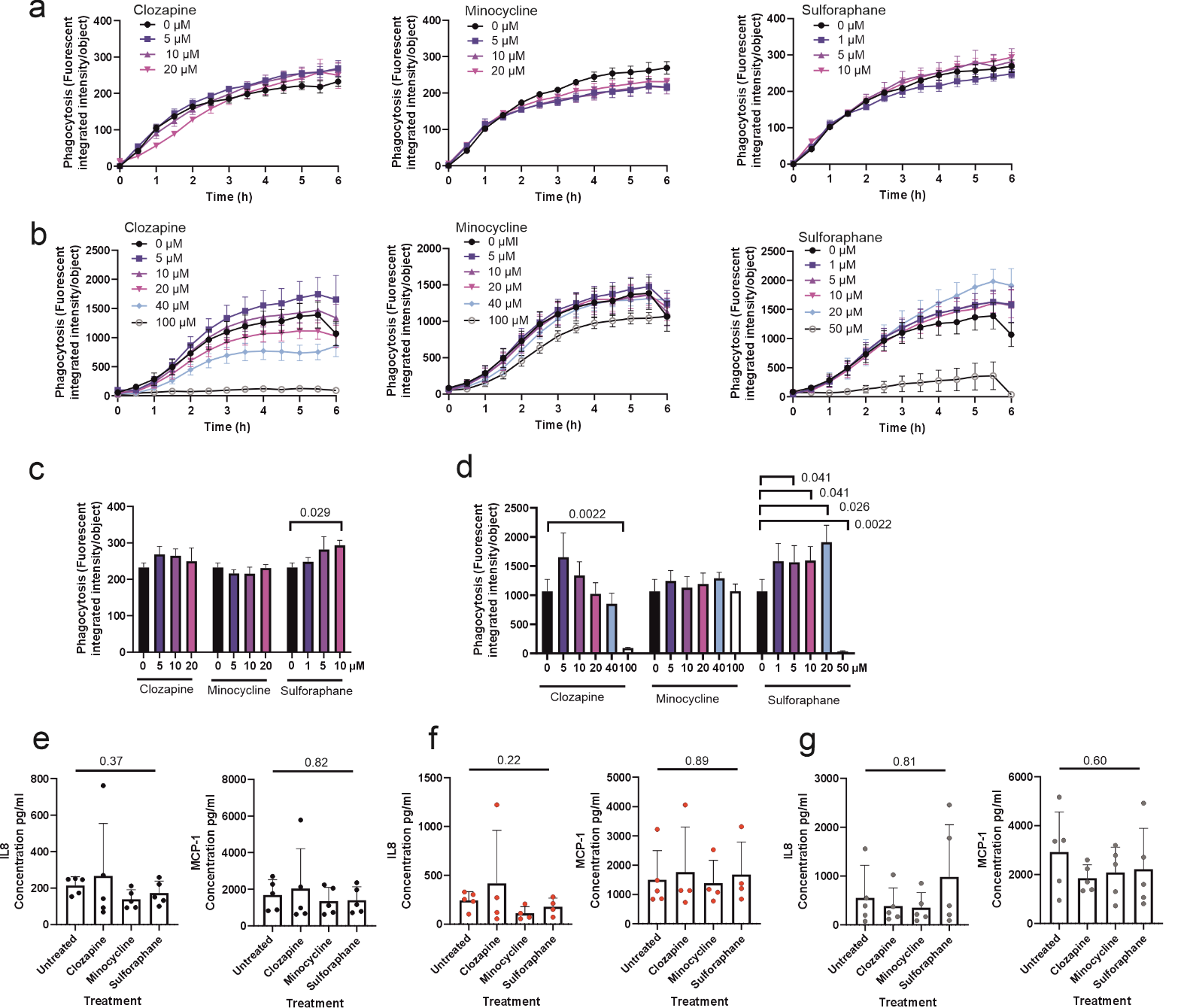
**

**Supplementary figure 10. Drug treatment effects on cytokine release and phagocytosis.** pHrodo zymosan phagocytosis during 6-hour imaging in **a)** CTRL iMGLs (clozapine, minocycline and sulforaphane) and **b)** CTRL iMφs (clozapine, minocycline and sulforaphane). Mean +/- SEM. n= 3 lines. Phagocytosis after 6 hours in **c)** CTRL iMGLs (U=0, p=0.0286) and **d)** CTRL iMφs (Clozapine: U=0, p=0.0022; Sulforaphane: U=5, p=0.0411; U=5, p=0.0411; U=4, p=0.0260; U=0, p=0.0022). Mann-Whitney test. IL8 and MCP1 secretion after 24-hour drug treatments in **e)** CTRL iMGLs (H(3)=3.160, p=0.3676; H(3)=0.9429, p=0.8151), **f)** ST iMGLs (H(3)=4.459, p=0.2234; H(3)=0.6784, p=0.8932), and **g)** HT iMGLs (H(3)=0.9771, p=0.8968; H(3)=1.857, p=0.6026). Kruskal-Wallis test. n= 4-5 lines per group.

**
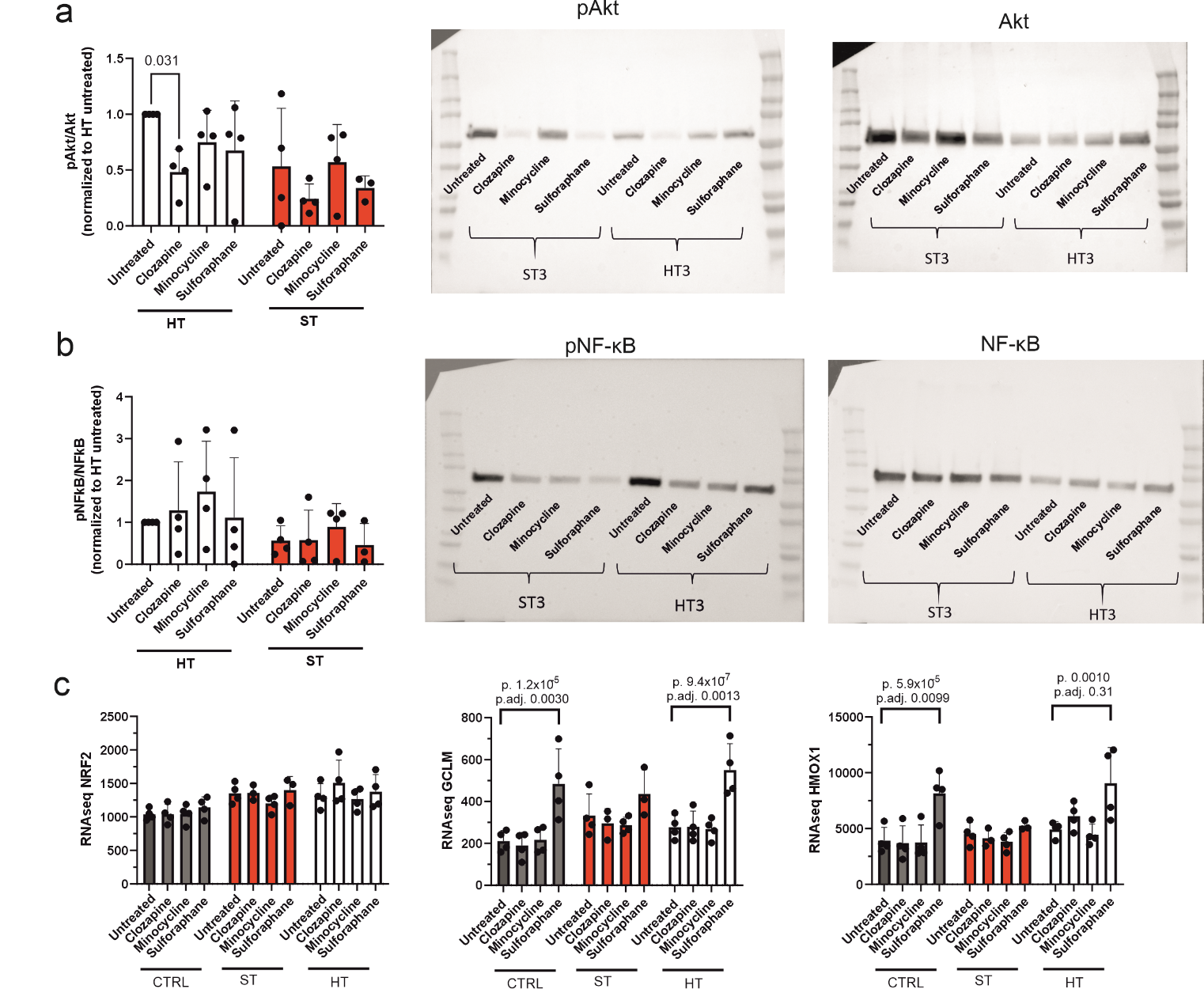
**

**Supplementary figure 11. NF-κB, Akt and NRF2 pathway activation in iMGLs after drug treatments.** **a)** phosphoAkt (pAkt) protein expression normalized to Akt expression, and pAkt and Akt western blot membrains. n=4 lines. (Untreated vs. Clozapine: 95% CI of diff.= 0.08483 to 0.9518, adj.p= 0.0311) **b)** phosphoNF-κB (pNF-κB) protein expression normalized to NF-κB expression, and pNF-κB p65 and NF-κB p65 western blot membrains. n=4 lines. Dunnett’s multiple comparisons tests were used for significance. **c)** NRF2 and its downstream pathway gene expression. RNAseq n=3-4 lines in each group.


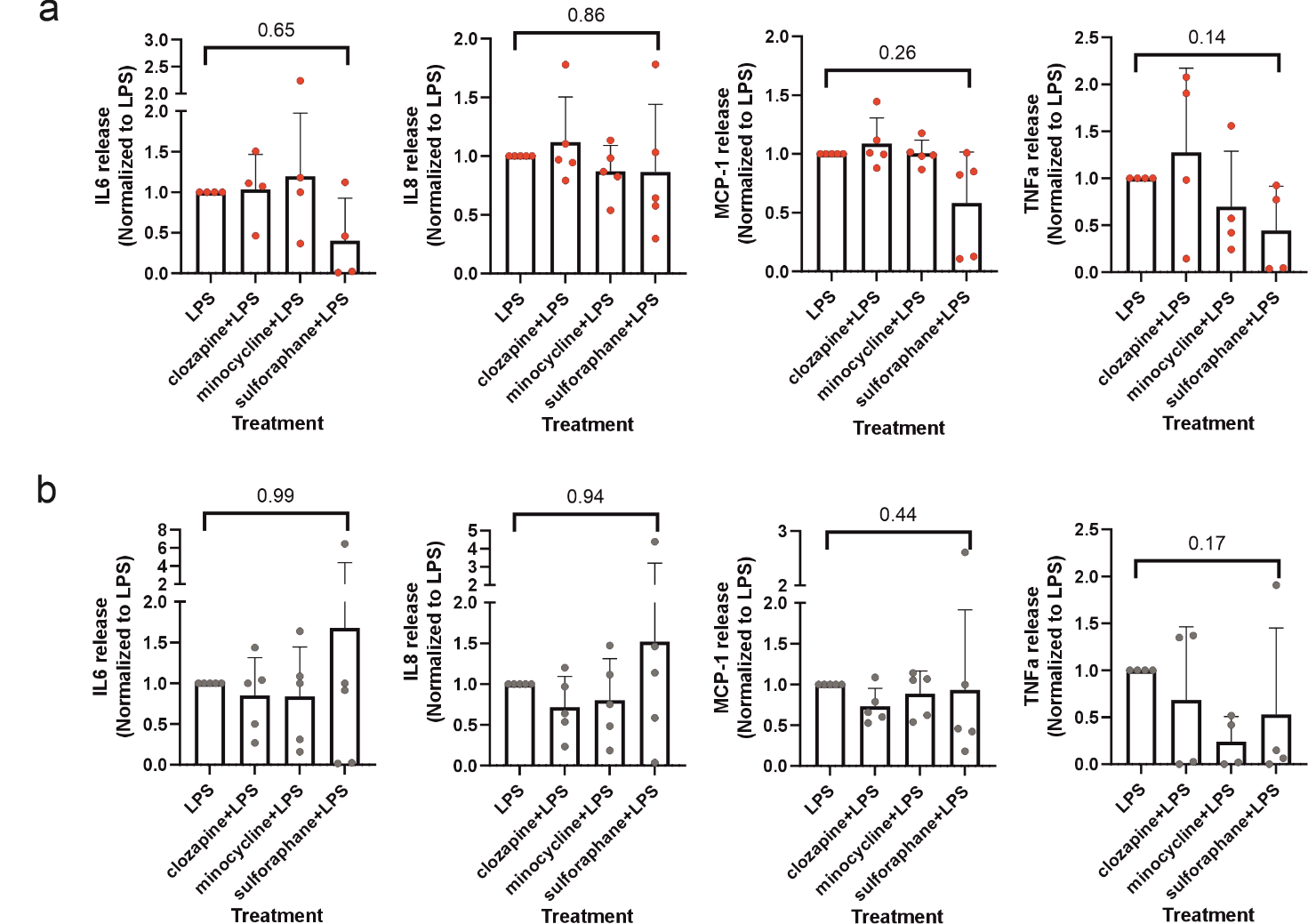


**Supplementary figure 12. Cytokine release after LPS in ST and HT twins.** Related to 6c. IL6, IL8, MCP1 and TNFa releases in **a)** ST iMGLs (χ2(4)=5.400, p=0.6489; χ2(4)=1.080, p=0.8566; χ2(4)=4.200, p=0.2603, χ2(4)=5.700, p=0.1411) or **b)** HT iMGLs (χ2(4)=0.300, p=0.9924; χ2(4)=0.6000, p=0.9438, χ2(4)=3.000, p=0.4446; χ2(4)=5.154, p=0.1723) after 30 min pre-treatment with 10 µM clozapine, 10 µM minocycline or 5 µM sulforaphane and 24-hour 100 ng/ml LPS treatment. Friedman test, n=4.
